# Supplementary material for: A versatile platform for chemical engineering of exosomes empowered by ADP-ribosyl cyclases
Source: Nat Commun. 2025 Dec 19;17:932. doi: 10.1038/s41467-025-67661-0 (PMC12830964; doi:10.1038/s41467-025-67661-0)
Supplement: Supplementary file 1 — Supplementary Information [file 41467_2025_67661_MOESM1_ESM.pdf]

A versatile platform for chemical engineering of exosomes empowered by ADP-ribosyl cyclases

Zhang and Singireddi *et al.*

Supplementary Table 1. Amino acid sequences of CD38-PDGFR TMD and CD9-CD38 fusion proteins. CD38 is shown in blue. PDGFR TMD and CD9 are colored green.

**Fusion protein name (Domain map)**	**Amino acid sequence**
CD38-PDGFR TMD fusion<br><br>(HA-CD38-G_4S-Myc-PDGFR TMD)	YPYDVPDYAGAQPARSRWRQQWSGPGT<br>TKRFPETVLARCVKYTEIHPEMRHVDCQ<br>SVWDAFKGAFISKHPCDITEEDYQPLMK<br>LGTQTVPCNKILLWSRIKDLAHQFTQVQ<br>RDMFTLEDTLLGYLADDLTWCGEFATSK<br>INYQSCPDWRKDCSNNPVSVFWKTVSR<br>RFAEAACDVVHVMLDGSRSKIFDKDSTF<br>GSVEVHNLQPEKVQTLEAWVIHGGRED<br>SRDLCQDPTIKELESIISKRNIQFSCKNIY<br>RPDKFLQCVKNPEDSSCTSEIGGGGSVD<br>EQKLISEEDLNAVGQDTQEVIVVPHSLPF<br>KVVVISAILALVVLTIISLIILIMLWQKKPR
CD9-CD38 fusion<br><br>(HA-CD9-G_4S-G_4S-CD38-FLAG)	YPYDVPDYAGAQPARSPVKGGTKCIKYL<br>LFGFNFIFWLAGIAVLAIGLWLRFDSQTK<br>SIFEQETNNNNSSFYTGVYILIGAGALM<br>MLVGFLGCCGAVQESQCMLGLFFGFLLV<br>IFAIEIAAAIWGYSHKDEVIKEVQEFYKD<br>TYNKLKTKDEPQRETLKAIHYALNCCGL<br>AGGVEQFISDICPKKDVLETFTVKSCPDA<br>IKEVFDNKFHIIGAVGIGIAVVMIFGMIFS<br>MILCCAIRRNREMVGGGGSGGGGSVDM<br>ANCEFSPVSGDKPCCRLSRRAQLCLGVSI<br>LVLILVVVLAVVVPRWRQQWSGPGTTKR<br>FPETVLARCVKYTEIHPEMRHVDCQSV<br>WDAFKGAFISKHPCDITEEDYQPLMKLG<br>TQTVPCNKILLWSRIKDLAHQFTQVQRD<br>MFTLEDTLLGYLADDLTWCGEFATSKIN<br>YQSCPDWRKDCSNNPVSVFWKTVSRRF<br>AEAACDVVHVMLDGSRSKIFDKDSTFGS<br>VEVHNLQPEKVQTLEAWVIHGGREDSR<br>DLCQDPTIKELESIISKRNIQFSCKNIYRP<br>DKFLQCVKNPEDSSCTSEIGGSDYKDDD<br>DK

Supplementary Table 2. Primer sequences.

Primer name	Sequence (5′-3′)
CD38-PDGFR Forward	GGCCAGATCTAGATGGAGGCAACAATGGTCAGG
CD38-PDGFR Reverse	GTTCGTCGACGCTTCCGCCCCCGCCGATCTCACTAG TACATGAACTATCCTCTGGGT
CD9-CD38 Forward 1	GTTCCAGATTATGCTGGGGCCCAGCCGGCCAGATCT CCGGTCAAAGGAGGCACCAAG
CD9-CD38 Reverse 1	CCCACCTCCACCGCTACCACCGCCTCCGACCATCTC GCGGTTCCTGC
CD9-CD38 Forward 2	GTCCGTCGACATGGCCAACTGCGAGTTCA
CD9-CD38 Reverse 2	TCGAGCGGCCGCCTTACTTGTCGTCAT

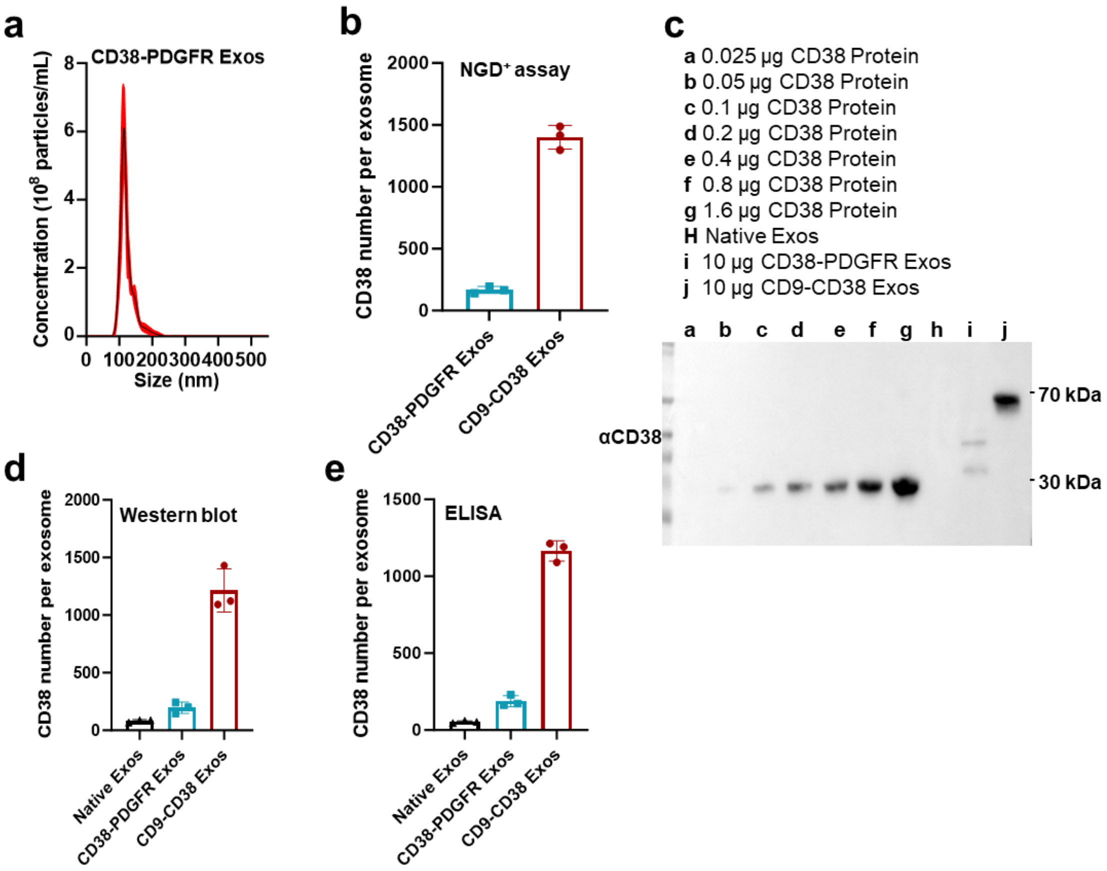

Supplementary Figure 1. Expression levels of CD38 on exosomes. (a) NTA analysis of purified CD38-PDGFR Exos (n = 6 per group). Data are shown as mean ± SD. (b) Numbers of CD38 per exosome as quantified by NGD$^+$-based enzymatic activity assays (n = 3 per group). Data are presented as mean ± SD. (c) Immunoblot analysis of CD38 expression levels on native exosomes, CD38-PDGFR Exos, and CD9-CD39 Exos as detected with an anti-CD38 antibody. Purified recombinant CD38 extracellular domain at varied amounts were included as standards. (d) and (e) Numbers of CD38 per exosome as quantified by immunoblot and ELISA assays (n = 3 per group). Data are shown as mean ± SD. Source data are provided as a Source Data file.

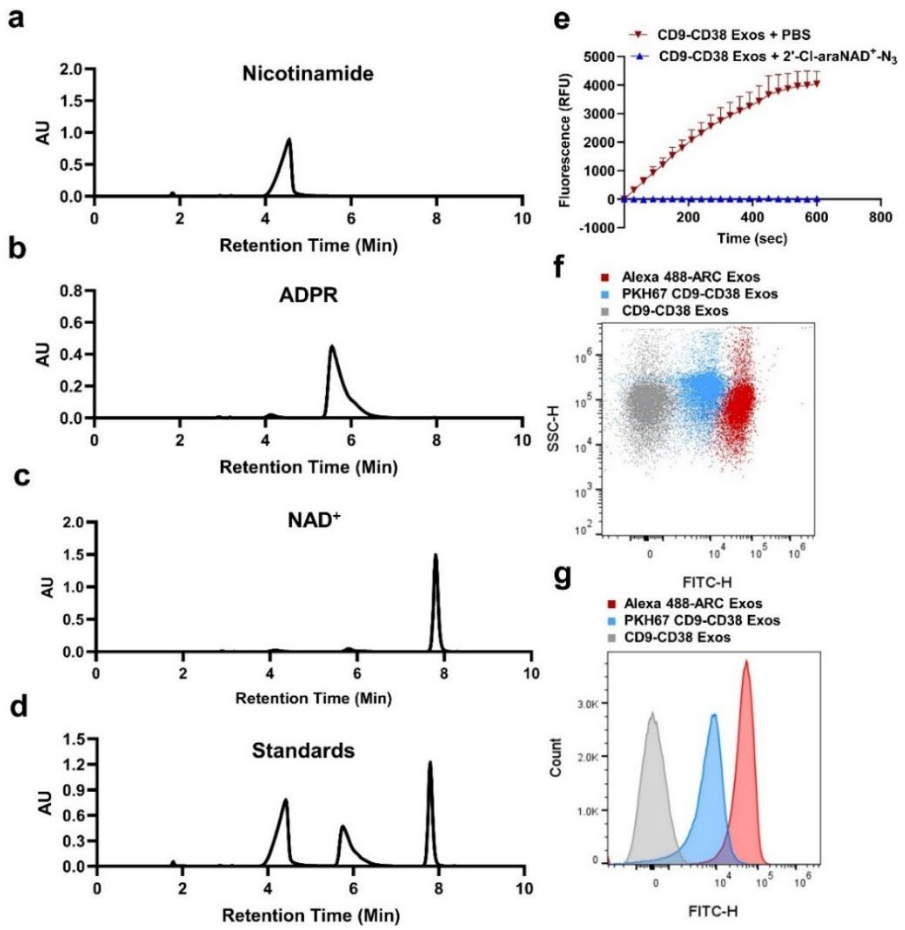

Supplementary Figure 2. HPLC analysis of standard compounds and nanoflow cytometry of Alexa 488-ARC Exos. (a) Nicotinamide. (b) ADPR. (c) NAD^+. (d) A mixture of nicotinamide, ADPR, and NAD^+. Compound(s) at 200 μM was injected into a C18 column for examination. (e) Enzymatic activity of 2′-Cl-araNAD^+-N_3-conjugated CD9-CD38 Exos. After conjugation with 2′-Cl-araNAD^+-N_3, CD9-CD38 Exos (10 μg mL^{-1}) were incubated with NGD^+ (100 μM) and CD38 catalytic activities were monitored by fluorescence signals at 410 nm. CD9-CD38 Exos without 2′-Cl-araNAD^+-N_3 conjugation were included as controls. (f) and (g) Nanoflow cytometric analysis of Alexa 488-ARC Exos by 2D dot plots (f) (side scatter (SSC) vs fluorescence intensity) and fluorescence histograms (g). PKH67-stained CD9-CD38 Exos (PKH67 CD9-CD38 Exos) and CD9-CD38 Exos were included as controls. Source data are provided as a Source Data file.

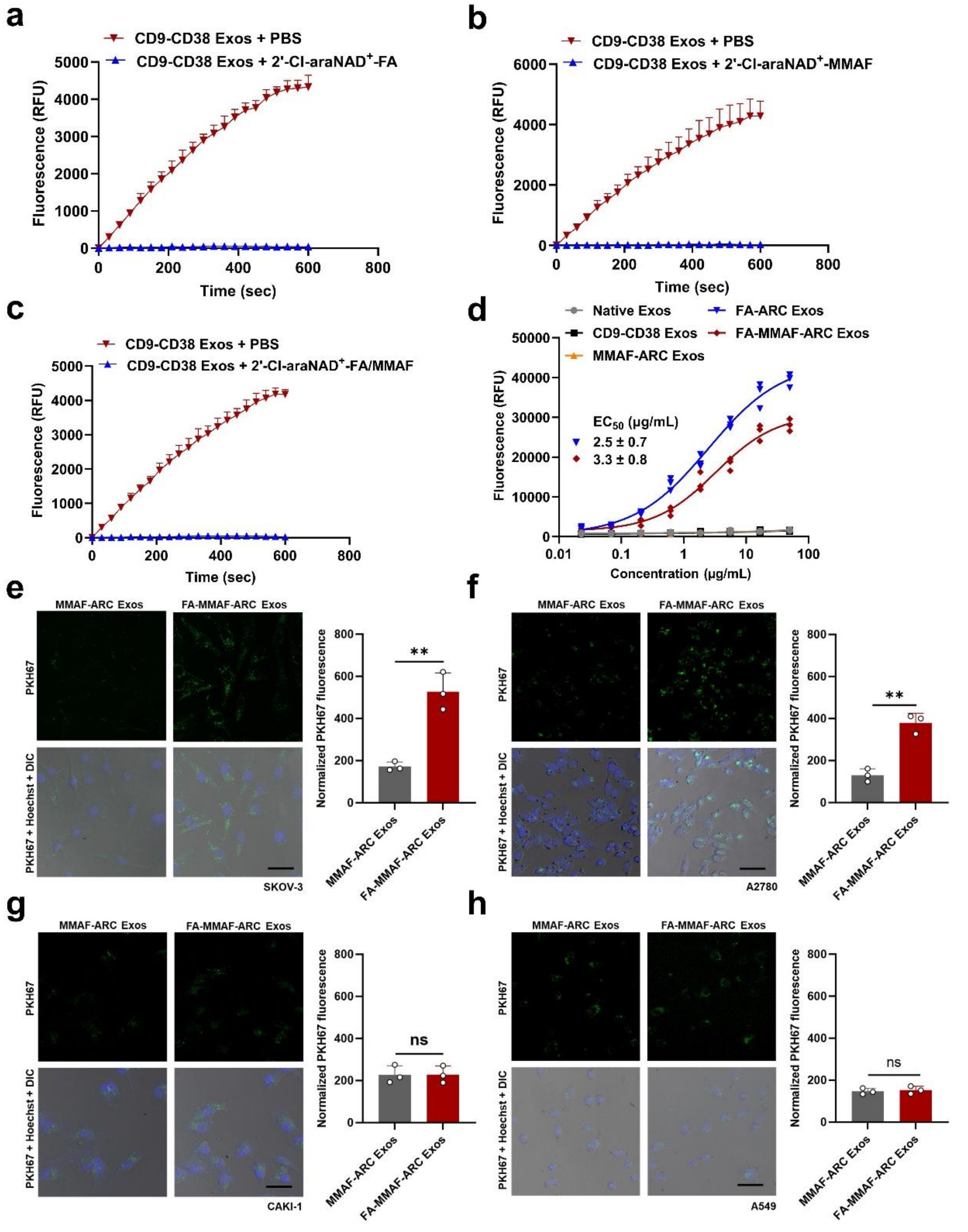

Supplementary Figure 3. Generation and characterization of FA-ARC Exos, MMAF-ARC Exos, and FA-MMAF-ARC Exos. (a)-(c) CD38 enzymatic activities of FA-ARC Exos (a), MMAF-ARC Exos (b), and FA-MMAF-ARC Exos (c). After conjugation with 2′-Cl-araNAD^{+}-FA, -MMAF, and -FA/MMAF, CD9-CD38 Exos were incubated with

NGD^+ and CD38 catalytic activities were monitored by fluorescence signals at 410 nm. CD9-CD38 Exos without conjugation were included as controls (n = 3 per group). Data are presented as mean ± SD. (d) Sandwich ELISA analysis of binding of FA-MMAF-ARC Exos to recombinant human FRα. Exosomes and anti-His_6 antibody were used as capture and detection reagents, respectively (n = 3 per group). Data are shown as mean ± SD. (e)-(h) Cellular uptake of MMAF-ARC Exos and FA-MMAF-ARC Exos by SKOV-3 (e), A2780 (f), CAKI-1 (g), and A549 (h) cells. PKH67-labeled exosomes were incubated with cells for 4 hour at 37°C. After fixation and staining with Hoechst, cells were imaged by confocal microscopy. PKH67 fluorescence intensities for each group were quantified and shown on right of each panel. Three biological replications were performed. Statistical analysis was performed using two-tailed unpaired student *t* tests. Data are presented as mean ± SD. Significance of finding was defined as follows, ns = not significant $p > 0.05$ and ** $p < 0.01$. Source data are provided as a Source Data file.

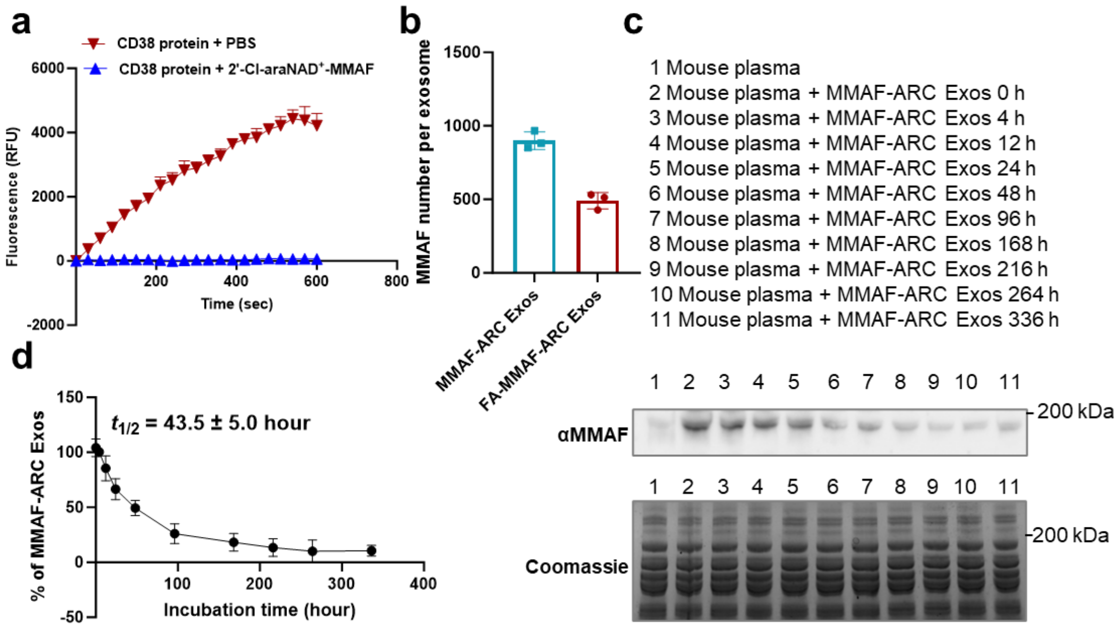

Supplementary Figure 4. Characterization of MMAF-ARC Exos and FA-MMAF-ARC Exos. (a) Enzymatic activity of MMAF-conjugated recombinant CD38 extracellular domain. After conjugation with 2′-Cl-araNAD$^+$-MMAF, CD38 protein was incubated with NGD$^+$ and its catalytic activity was monitored by fluorescence signals at 410 nm. CD38 without 2′-Cl-araNAD$^+$-MMAF conjugation was included as controls (n = 3 per group). Data are presented as mean ± SD. (b) Numbers of MMAF per exosome as quantified by ELISA assays (n = 3 per group). Data are shown as mean ± SD. (c) and (d) Stability of MMAF-ARC Exos in mouse plasma as examined by immunoblots with an anti-MMAF antibody (c) and quantitative analysis of intact band intensities (d) (n = 3 per group). Data are presented as mean ± SD. Source data are provided as a Source Data file.

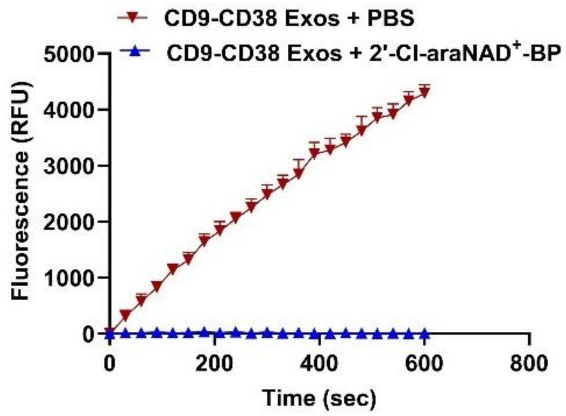

Supplementary Figure 5. CD38 enzymatic activity of BP-ARC Exos. After conjugation with 2′-Cl-araNAD$^+$-BP, CD9-CD38 Exos were incubated with NGD$^+$ and CD38 catalytic activities were monitored by fluorescence signals at 410 nm. CD9-CD38 Exos without conjugation were included as controls (n = 3 per group). Data are presented as mean ± SD. Source data are provided as a Source Data file.

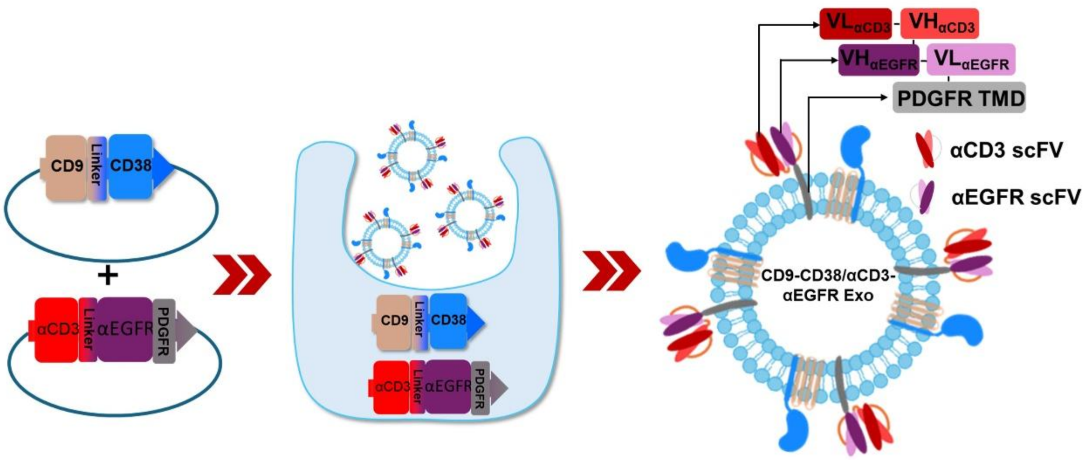

Supplementary Figure 6. Schematic of the design and generation of CD9-CD38/αCD3-αEGFR Exos.

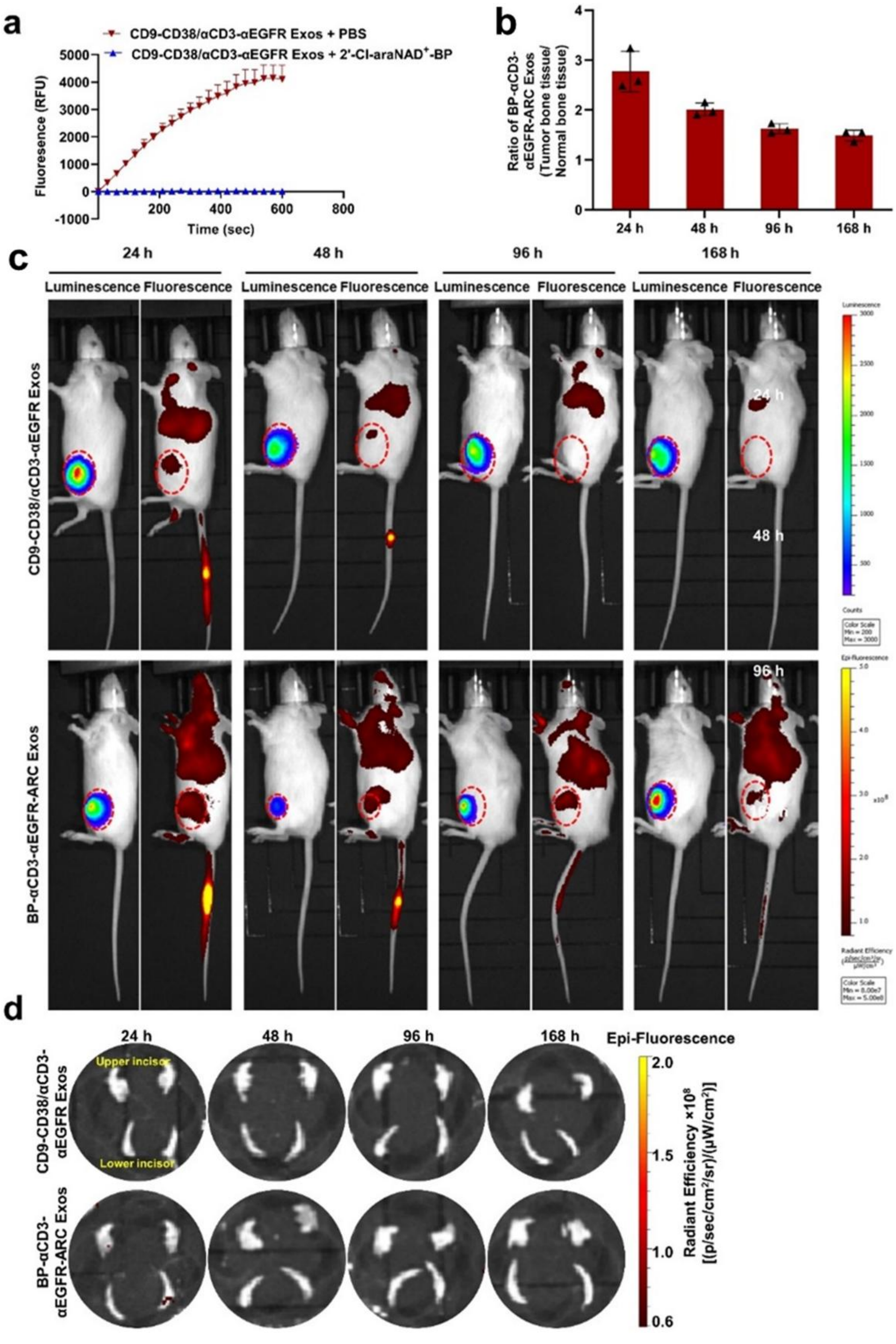

Supplementary Figure 7. CD38 enzymatic activity and biodistribution of BP-αCD3-αEGFR-ARC Exos. (a) After conjugation with 2′-Cl-araNAD$^+$-BP, CD9-CD38/αCD3-αEGFR Exos were incubated with NGD$^+$ and CD38 catalytic activities were monitored by

fluorescence signals at 410 nm. CD9-CD38/αCD3-αEGFR Exos without conjugation were included as controls (n = 3 per group). Data are shown as mean ± SD. (b) Biodistribution ratio of BP-αCD3-αEGFR-ARC Exos for bone tumor tissue to normal bone tissue at 24-168 h (n = 3 mice per group). Data are presented as mean ± SD. (c) Biodistribution of BP-αCD3-αEGFR-ARC Exos in mice bearing EGFR-positive BT-20 tumors. Mice with luciferase-expressing BT-20 tumors implanted in left hindlimbs via para-tibial injections received single dose of DiR-labeled exosomes and were imaged for luminescence and fluorescence at 24-168 h. (d) Biodistribution of BP-αCD3-αEGFR-ARC Exos in teeth of mice bearing BT-20 tumors at 24-168 h. Source data are provided as a Source Data file.

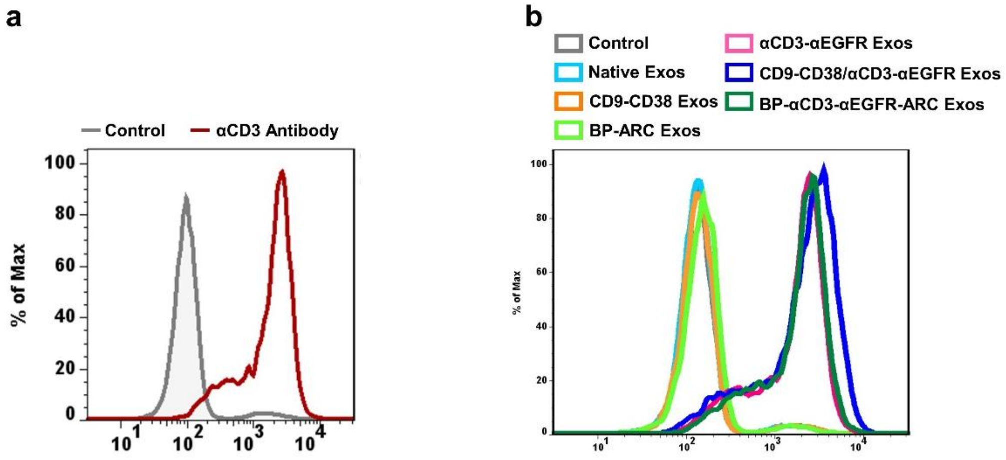

Supplementary Figure 8. Flow cytometry of CD3 expression and exosome binding to Jurkat cells. (a) CD3 expression levels on Jurkat cells. (b) Binding of BP-αCD3-αEGFR-ARC Exos to Jurkat cells.

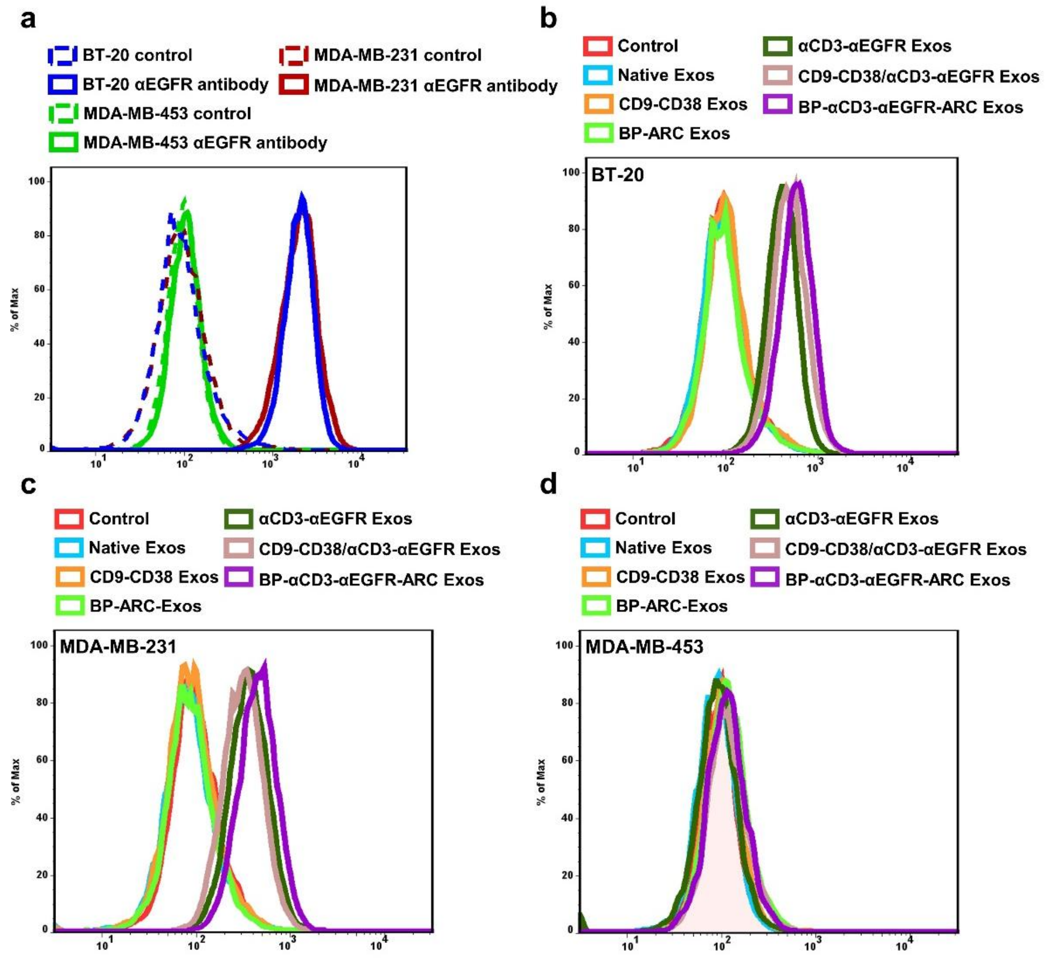

Supplementary Figure 9. Flow cytometry of EGFR expression and exosome binding to breast cancer cells. (a) EGFR expression levels on BT-20, MDA-MB-231, and MDA-MB-453 cells. (b)-(d) Binding of BP-αCD3-αEGFR-ARC Exos to BT-20 (b), MDA-MB-231 (c), and MDA-MB-453 (d) cells.

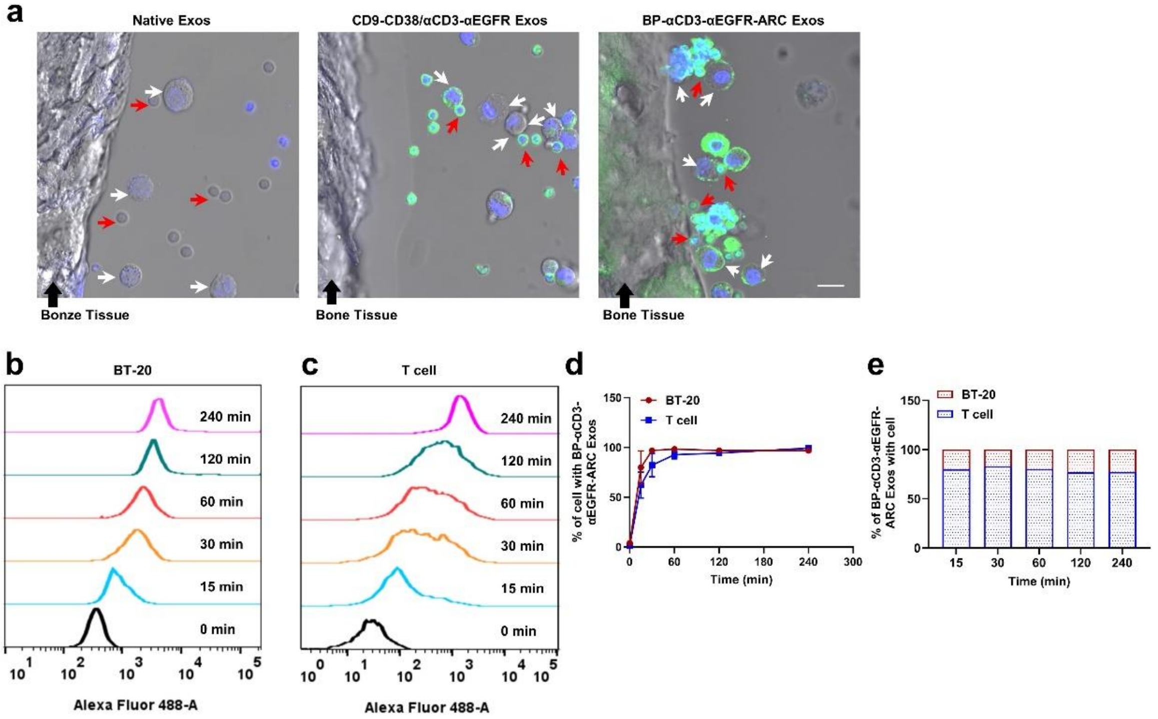

Supplementary Figure 10. Analysis of binding of BP-αCD3-αEGFR-ARC Exos. (a) Confocal images of binding of PKH67-labeled exosomes to T cells (red arrows), BT-20 cells (white arrows), and bone tissues (left side of each image). Non-decalcified bone sections were incubated with BT-20 and T cells for 30 min at 4°C in the presence of PKH67-labeled native exosomes, CD9-CD38/αCD3-αEGFR Exos, or BP-αCD3-αEGFR-ARC Exos, followed by fixation and staining with Hoechst for confocal microscopy. Scale bars, 20 μm. Experiments in Supplementary Figure 10a were repeated independently three times with similar results. (b)-(e) Flow cytometry of binding of BP-αCD3-αEGFR-ARC Exos to BT-20 (b) and T cells (c) and quantitative analysis of binding efficiency (d) and proportion (e) of BP-αCD3-αEGFR-ARC Exos to BT-20 and T cells. T cells (4×10^5 cells mL^{-1}) and BT-20 cells (5×10^4 cells mL^{-1}) were mixed, incubated with PKH67-labeled BP-αCD3-αEGFR-ARC Exos for 0-240 min at 37°C, and then analyzed by flow cytometry (n = 3 per group). Data are presented as mean ± SD. Source data are provided as a Source Data file.

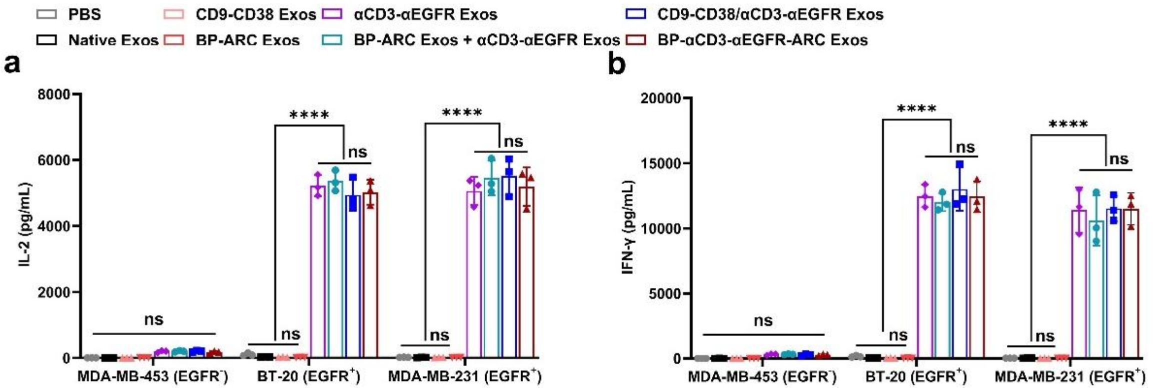

Supplementary Figure 11. EGFR-dependent activation of human T cells by BP-αCD3-αEGFR-ARC Exos. (a) Levels of secreted IL-2. (b) Levels of released IFN-γ. Non-activated human PBMCs (effector cells) were incubated with MDA-MB-453 ($EGFR^-$), BT-20 ($EGFR^+$), and MDA-MB-231 ($EGFR^+$) target cells (E:T ratio: 8) in the presence of PBS or exosomes (1 μg mL^{-1}) for 48 h, followed by measurements of released IL-2 and IFN-γ with ELISA (n = 3 per group). Three biological replications were performed. Data are presented as mean ± SD. Statistical analysis was performed using ordinary one-way ANOVA with Tukey's multiple comparison test. Significance of finding was defined as follows, ns = not significant $p > 0.05$ and **** $p < 0.0001$. Source data are provided as a Source Data file.

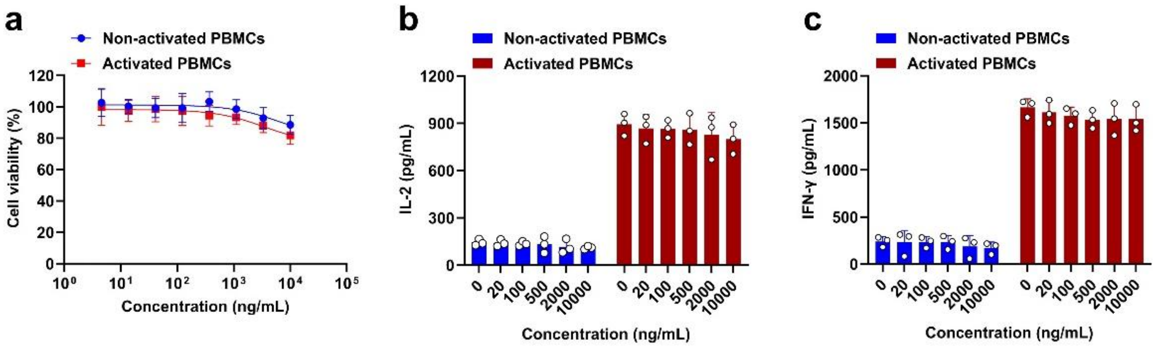

Supplementary Figure 12. Effects of CD9-CD38 Exos on functions of human PBMCs. (a) Cell viabilities of non-activated and activated PBMCs following incubation with CD9-CD38 Exos (n = 3 per group). Data are presented as mean ± SD. (b) and (c) Levels of IL-2 (b) and IFN-γ (c) secreted by non-activated and activated PBMCs after incubation with CD9-CD38 Exos. Human PBMCs were activated with αCD3 antibody (10 µg mL^{-1}) and αCD28 antibody (2.5 µg mL^{-1}). Non-activated and activated PBMCs were incubated with CD9-CD38 Exos at various concentrations for 48 h at 37°C, followed by measurements of cell viabilities and released cytokine concentrations (n = 3 per group). Data are shown as mean ± SD. Source data are provided as a Source Data file.

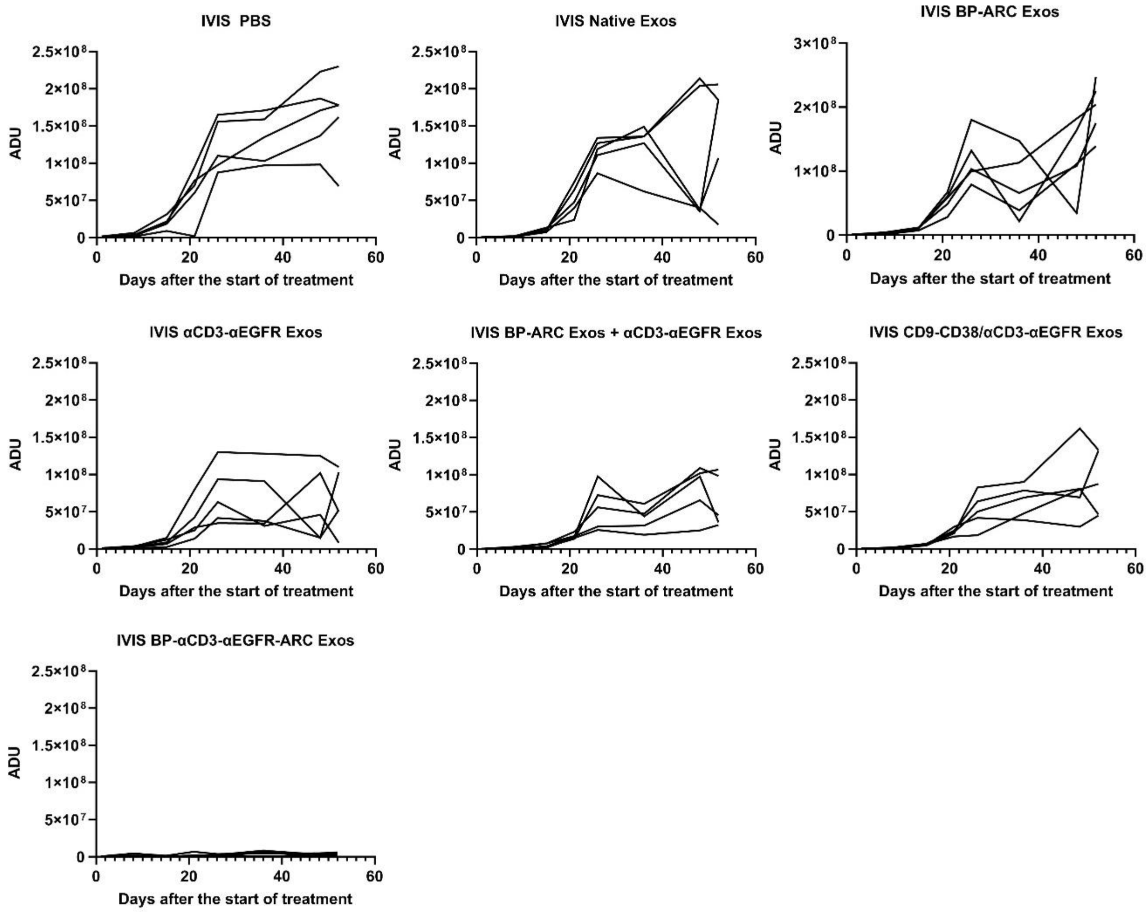

Supplementary Figure 13. Bioluminescence intensities of individual BT-20 tumor-bearing mice after the start of treatment. Source data are provided as a Source Data file.

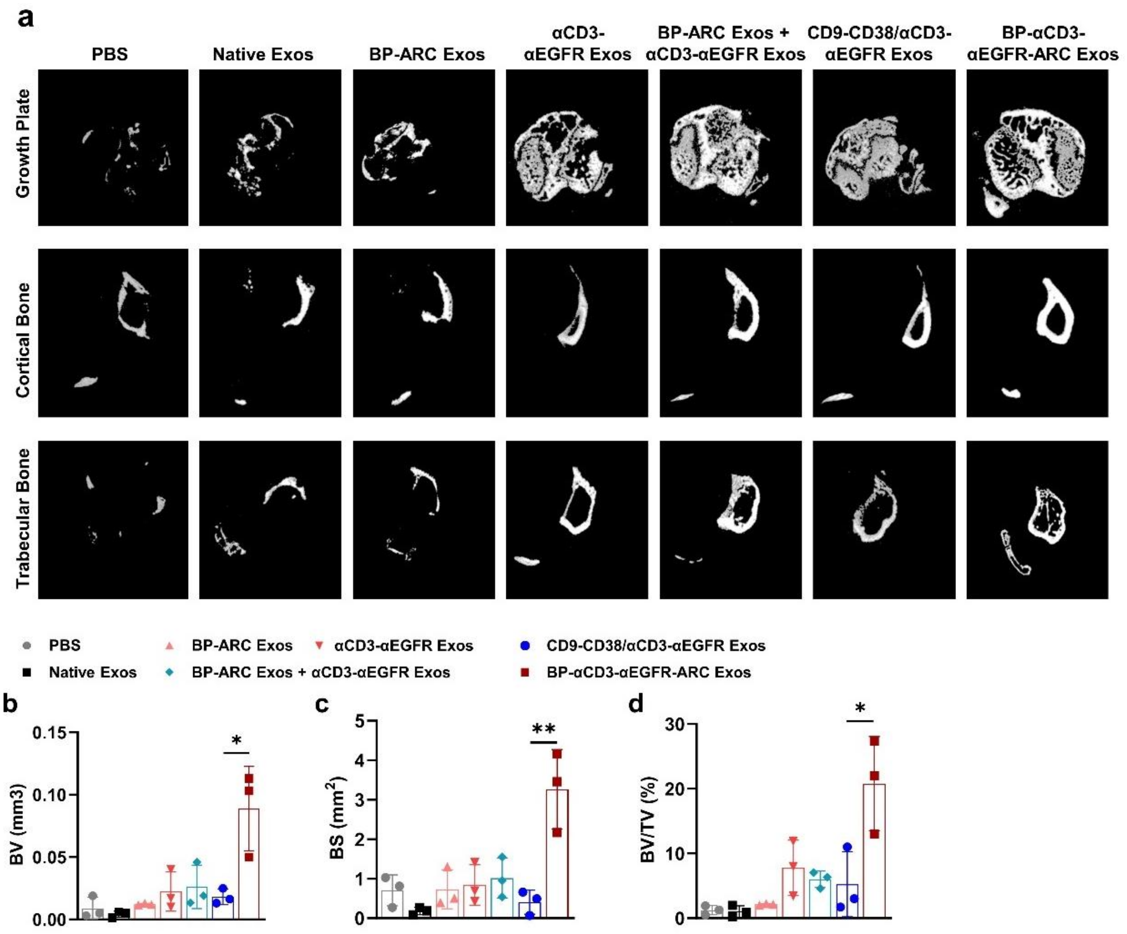

Supplementary Figure 14. Micro-CT analysis of left hindlimb bones of BT-20 tumor-bearing mice at the end of anti-tumor efficacy and toxicity study. (a) Representative slices from each group. Top panel: slices near growth plates. Middle panel: cortical bones (3.25 mm distal of growth plates). Bottom panel: trabecular bones (1.25 mm distal of growth plates). (b)-(d) Bone volume (BV) (b), bone surface (BS) (c), and bone volume fraction (BV/total volume (TV)) (d) of parts of trabecular bones from left hindlimbs for each group of mice as quantified by micro-CT scanning (n = 3 per group). Data are presented as mean ± SD. Statistical analysis was performed using ordinary one-way ANOVA with Dunnett's multiple comparison test. Significance of finding was defined as follows, * $p < 0.05$ and ** $p < 0.01$. Source data are provided as a Source Data file.

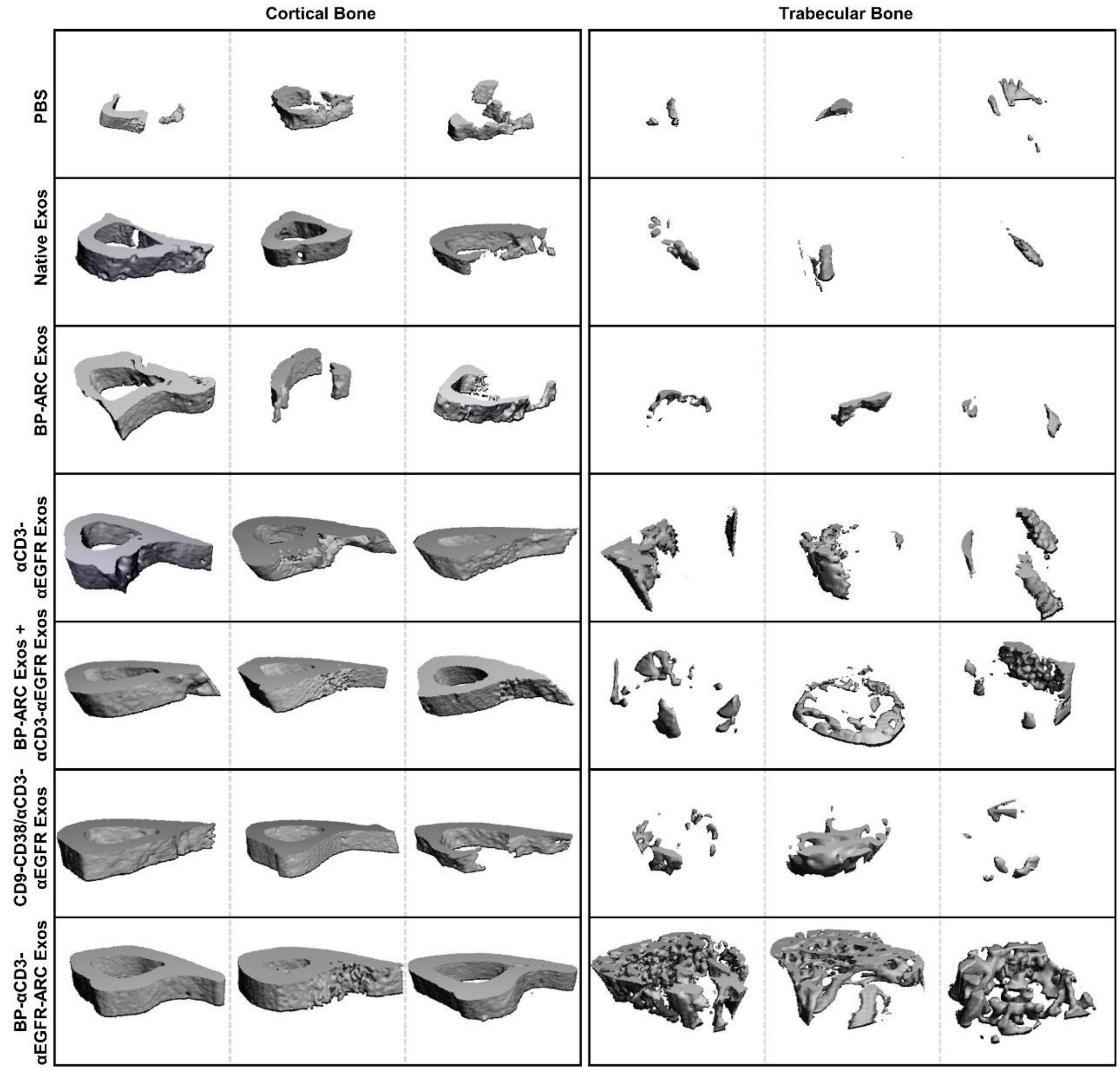

Supplementary Figure 15. Micro-CT 3D images of left hindlimb bones of BT-20 tumor-bearing mice at the end of anti-tumor efficacy and toxicity study (n = 3 per group). Left panel: cortical bones. Right panel: trabecular bones.

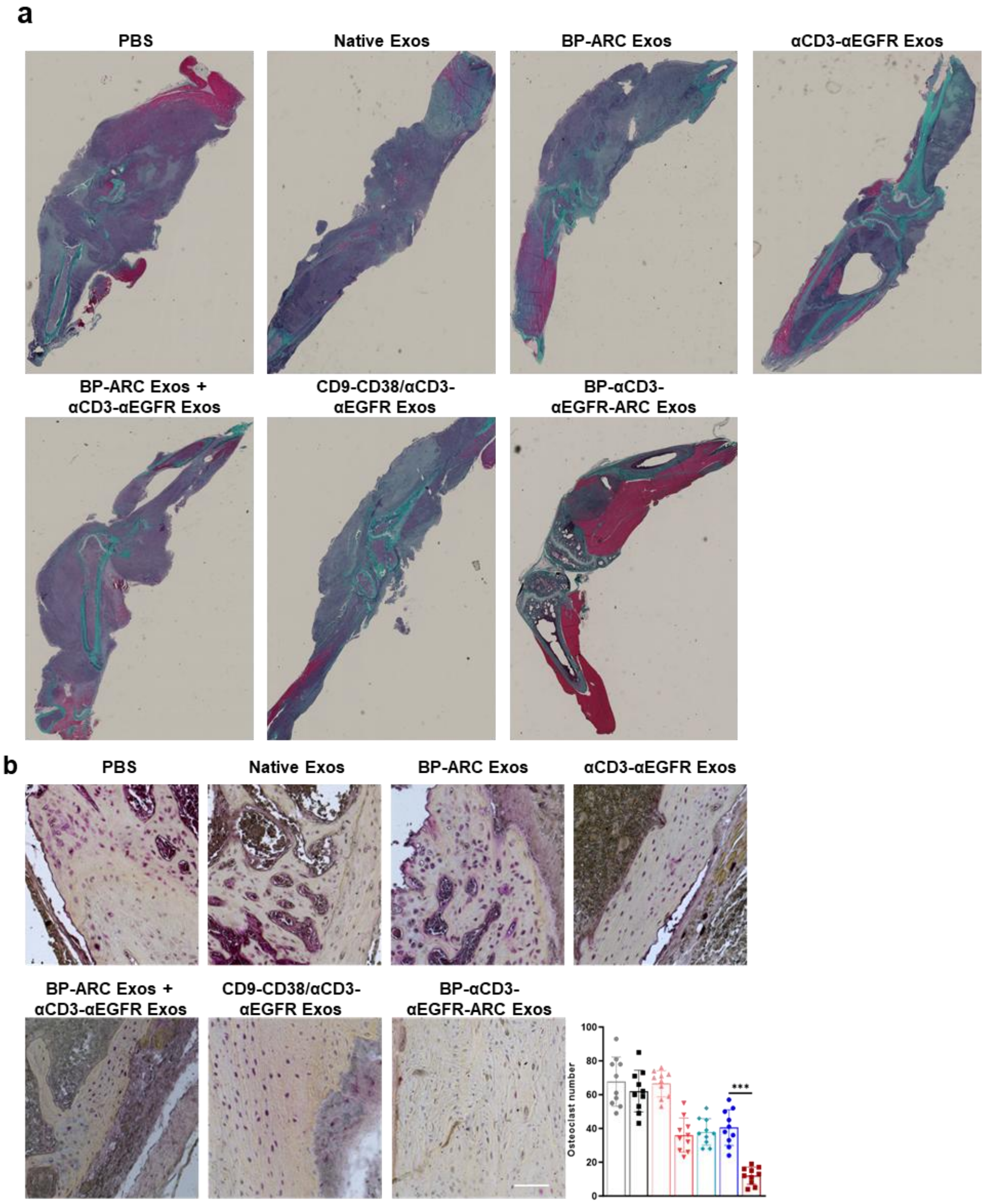

Supplementary Figure 16. Representative Masson-Goldner trichrome- and TRAP-stained images of left hindlimb sections from all BT-20 tumor-bearing mice groups. (a) Representative Masson-Goldner trichrome-stained images collected from each group. (b) Representative TRAP-stained images collected from each group. Scale bars, 100 μm. Numbers of osteoclasts were counted for each treatment group (n = 10 per group). Data are presented as mean ± SD. Statistical analysis was performed using ordinary one-way ANOVA with Dunnett's multiple comparison test. Significance of finding was defined as follows, *** $p < 0.001$. Source data are provided as a Source Data file.

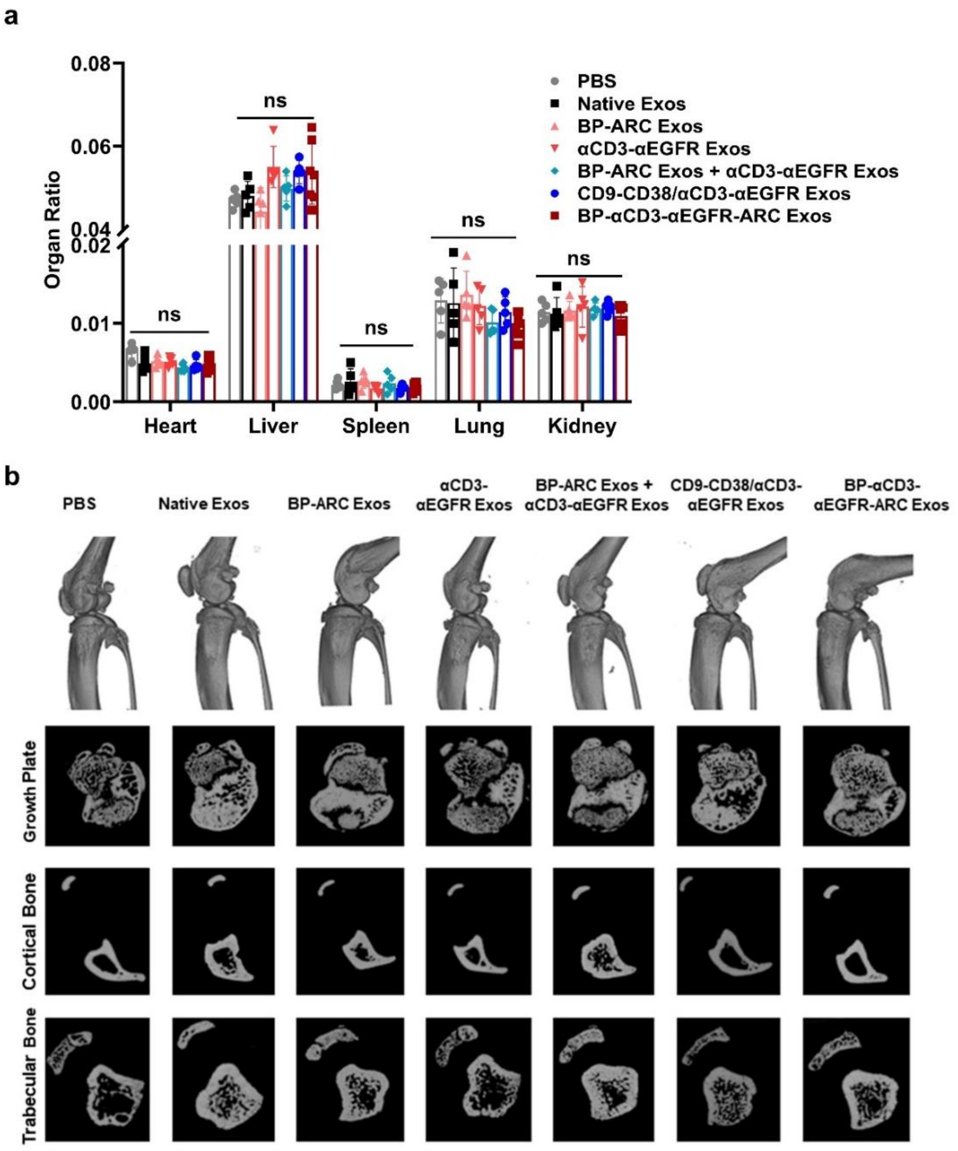

Supplementary Figure 17. *In vivo* toxicity study of BP-αCD3-αEGFR-ARC Exos. (a) Weight ratios of major organs for each group of mice bearing BT-20 tumors at the end of anti-tumor efficacy study (n = 5 or 7 per group). Data are shown as mean ± SD. Statistical analysis was performed using ordinary one-way ANOVA with Dunnett's multiple comparison test. Significance of finding was defined as follows, ns = not significant $p > 0.05$. (b) Representative micro-CT images of right hindlimbs (upper panel) and growth plates, cortical bones, and trabecular bones of the right hindlimbs (lower panel) for each group at the end of the study. Source data are provided as a Source Data file.

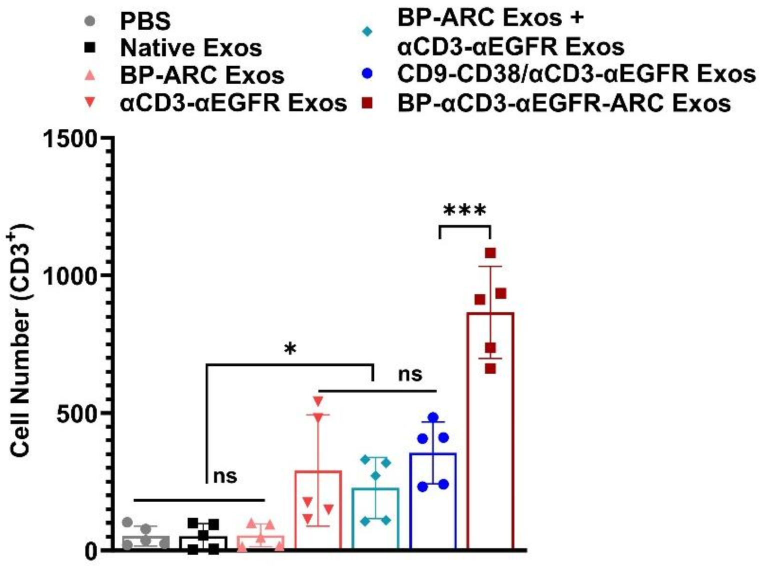

Supplementary Figure 18. Quantitative analysis of infiltrating T cells in BT-20 tumors at the end of anti-tumor efficacy and toxicity study by flow cytometry (n = 5 per group). Data are shown as mean ± SD. Statistical analysis was performed using ordinary one-way ANOVA with Tukey's multiple comparison test. Significance of finding was defined as follows, ns = not significant $p > 0.05$, * $p < 0.05$, and *** $p < 0.001$. Source data are provided as a Source Data file.

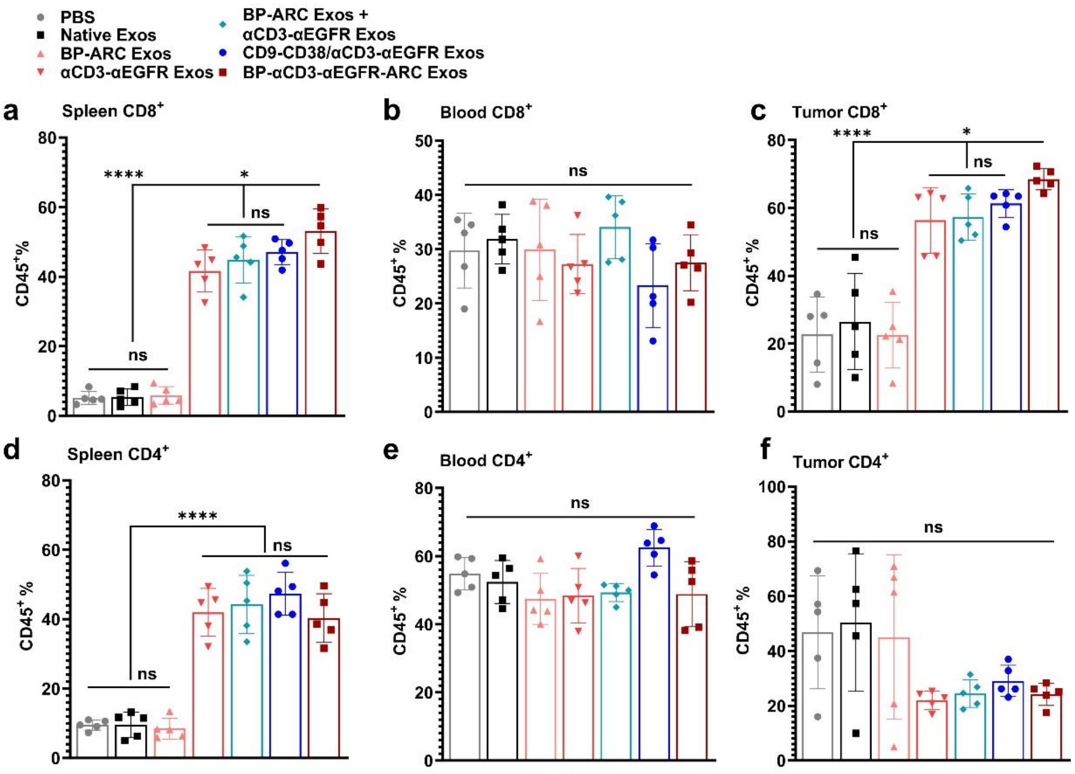

Supplementary Figure 19. T-cell infiltration for BT-20 tumor-bearing mice at the end of anti-tumor efficacy and toxicity study by flow cytometry. (a)-(f) Percentages of $CD8^+$ (a)-(c) and $CD4^+$ (d)-(f) T cells in $CD45^+$ cells in spleens (a),(d), blood (b),(e), and tumors (c),(f) for each group of mice at the end of study ($n = 5$ per group). Data are presented as mean ± SD. Statistical analysis was performed using ordinary one-way ANOVA with Tukey's multiple comparison test. Significance of finding was defined as follows, ns = not significant $p > 0.05$, * $p < 0.05$, and **** $p < 0.0001$. Source data are provided as a Source Data file.

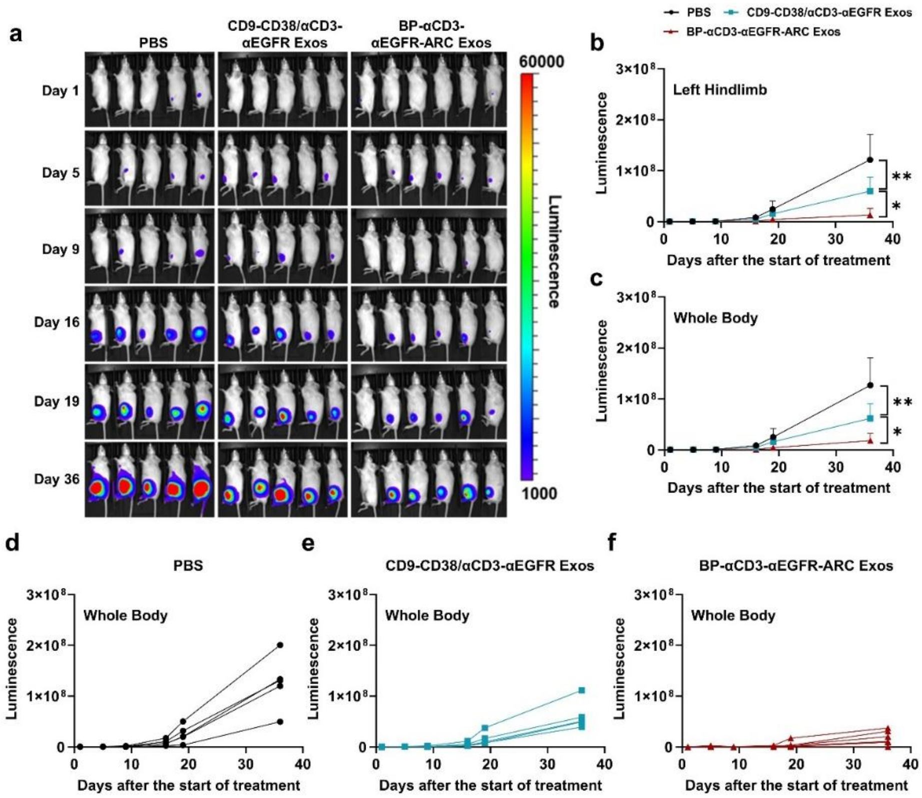

Supplementary Figure 20. *In vivo* therapeutic efficacy of BP-αCD3-αEGFR-ARC Exos for mice implanted with MDA-MB-231 cells. The left hindlimbs of NSG mice (n = 5 or 6 per group) were inoculated with MDA-MB-231 cells via para-tibial injections. Human PBMCs were intravenously injected on days 7 and 13 after tumor implantation. One day after the first PBMCs injections, mice were treated (i.v.) with PBS, CD9-CD38/αCD3-αEGFR Exos (10 mg kg^{-1}), or BP-αCD3-αEGFR-ARC Exos (10 mg kg^{-1}) every other day for six times. (a) Bioluminescence images of mice from all groups after the start of treatment. (b) and (c) Bioluminescence intensities of left hindlimbs (b) and whole body (c) for each group of mice after the start of treatment. Data are presented as mean ± SEM. Statistical analysis was performed using one-way ANOVA with the Geisser-Greenhouse correction and Dunnett's multiple comparison test for (b) and (c). Significance of finding was defined as follows: * $p < 0.05$ and ** $p < 0.01$. (d)-(f) Whole-body bioluminescence intensities of individual mice for PBS (d), CD9-CD38/αCD3-αEGFR Exos (e), and BP-αCD3-αEGFR-ARC Exos (f) groups after the start of treatment. Source data are provided as a Source Data file.

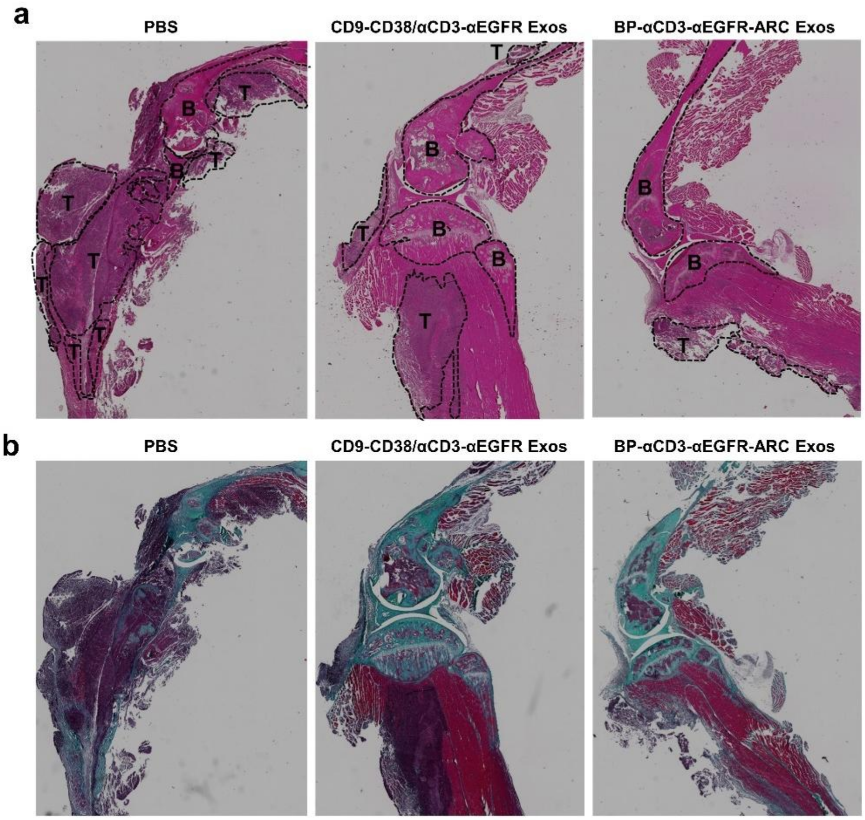

Supplementary Figure 21. Representative H&E- and Masson-Goldner trichrome-stained images of left hindlimb sections of mice bearing MDA-MB-231 tumors at the end of anti-metastasis study. (a) Representative H&E-stained images from each group. (b) Representative Masson-Goldner trichrome-stained images from each group. T: tumor. B: bone. Experiments in Supplementary Figure 21 were repeated independently three times with similar results.

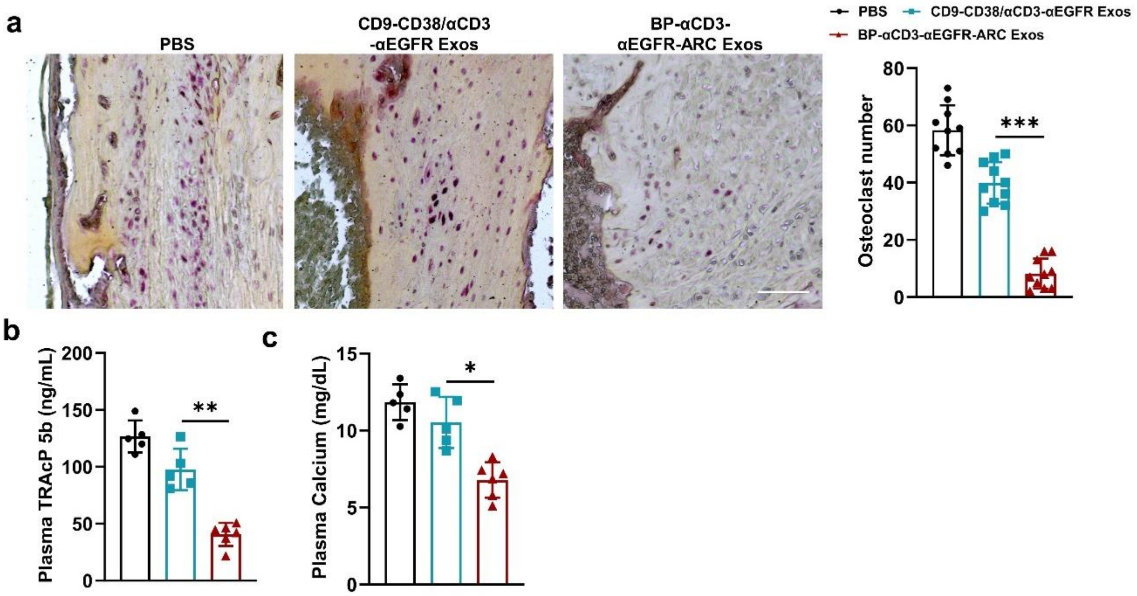

Supplementary Figure 22. Representative TRAP-stained images of left hindlimb sections and plasma levels of TRAcP 5b and calcium for each group of mice bearing MDA-MB-231 tumors at the end of anti-metastasis study. (a) Representative TRAP-stained images from each group. Numbers of osteoclasts were counted for each group (n = 10 per group). Data are presented as mean ± SD. Scale bars, 100 µm. (b) Plasma levels of TRAcP 5b at the end of anti-metastasis study (n = 5 or 6 per group). (c) Plasma levels of calcium at the end of anti-metastasis study (n = 5 or 6 per group). Experiments in Supplementary Figure 22a were repeated independently three times with similar results. Statistical analysis was performed using ordinary one-way ANOVA with Dunnett's multiple comparison test. Significance of finding was defined as follows, * $p < 0.05$, ** $p < 0.01$, and *** $p < 0.001$. Source data are provided as a Source Data file.

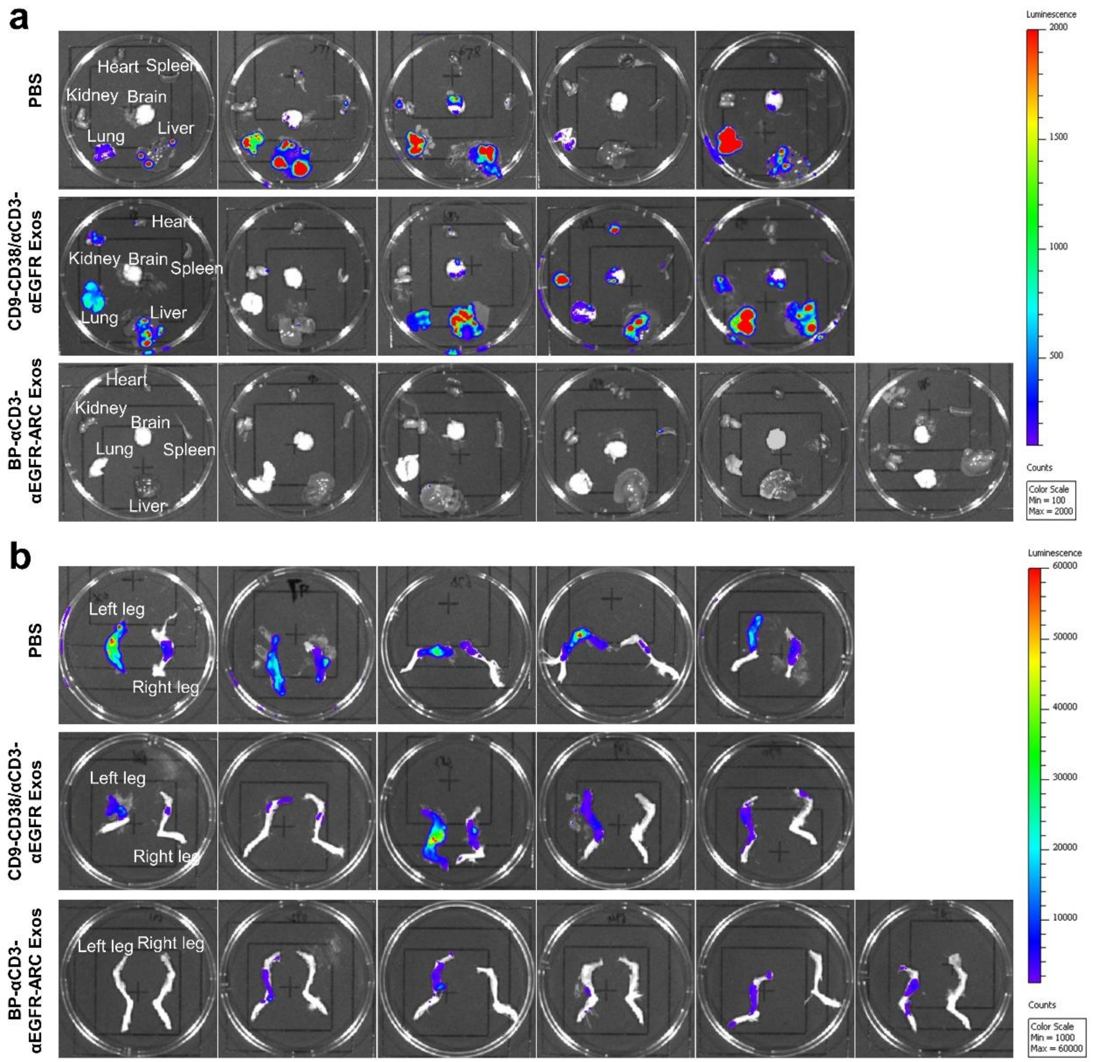

Supplementary Figure 23. Luminescent images of major organs and hindlimbs for each group of mice bearing MDA-MB-231 tumors at the end of anti-metastasis study. (a) Luminescent images of major organs for each group (n = 5 or 6 per group). (b) Luminescent images of hindlimbs for each group (n = 5 or 6 per group).

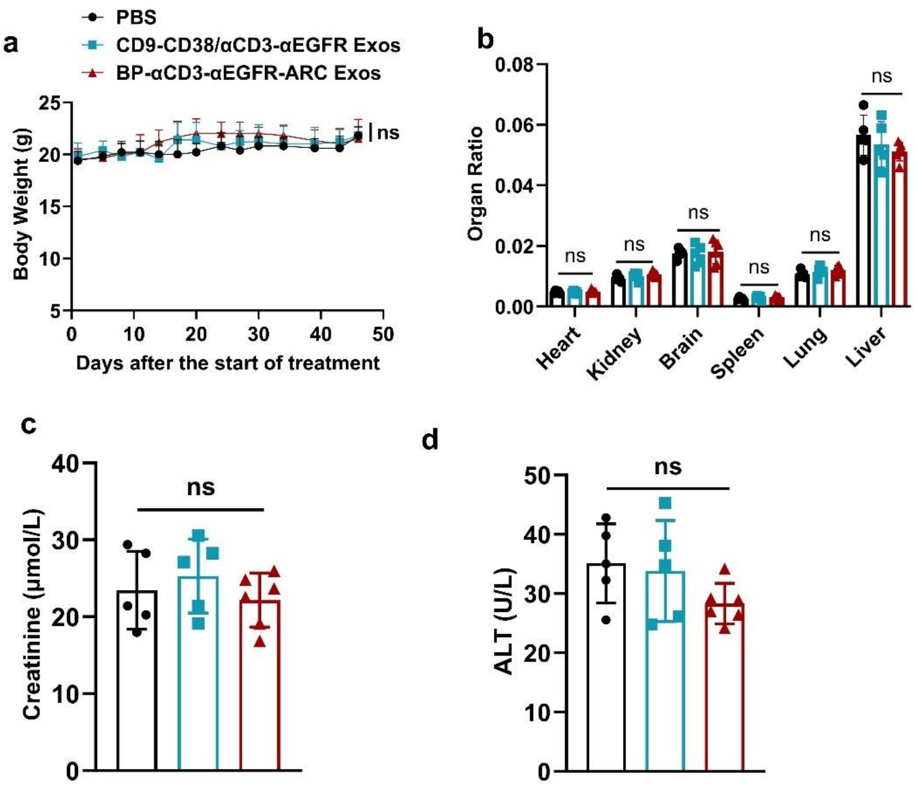

Supplementary Figure 24. *In vivo* toxicity of BP-αCD3-αEGFR-ARC Exos for MDA-MB-231 tumor-bearing mice. (a) Average body weights for each group of mice during the treatment study (n = 5 or 6 per group). (b) Weight ratios of major organs for each group of mice at the end of study (n = 5 or 6 per group). (c) and (d) Plasma levels of creatinine (c) and ALT (d) for each group of mice at the end of study (n = 5 or 6 per group). Data are shown as mean ± SD. Statistical analysis was performed using ordinary one-way ANOVA with Dunnett's multiple comparison test. Significance of finding was defined as follows, ns = not significant $p > 0.05$. Source data are provided as a Source Data file.

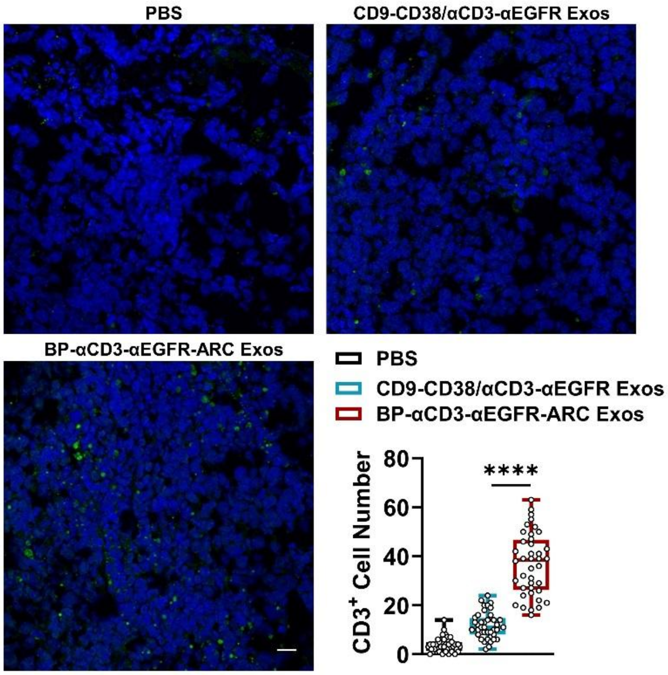

Supplementary Figure 25. Representative immunohistofluorescence images of tumor sections and quantitative analysis of tumor-infiltrating T cells for each group of mice bearing MDA-MB-231 tumors at the end of anti-metastasis study. Blue: nuclei stained with DAPI. Green: $CD3^+$ T cells stained with the anti-human CD3 antibody. Scale bars: 50 μm. Numbers of T cells were quantified with 20 fields of view per region and two mice per group. Data are shown in box-and-whisker plots with the line in the box as median and the lower and upper edge of the box as first and third quartile, respectively. Whiskers extending from the box represent the overall spread of the data. Statistical analysis was performed using ordinary one-way ANOVA with Dunnett's multiple comparison test. Significance of finding was defined as follows, **** $p < 0.0001$. Source data are provided as a Source Data file.

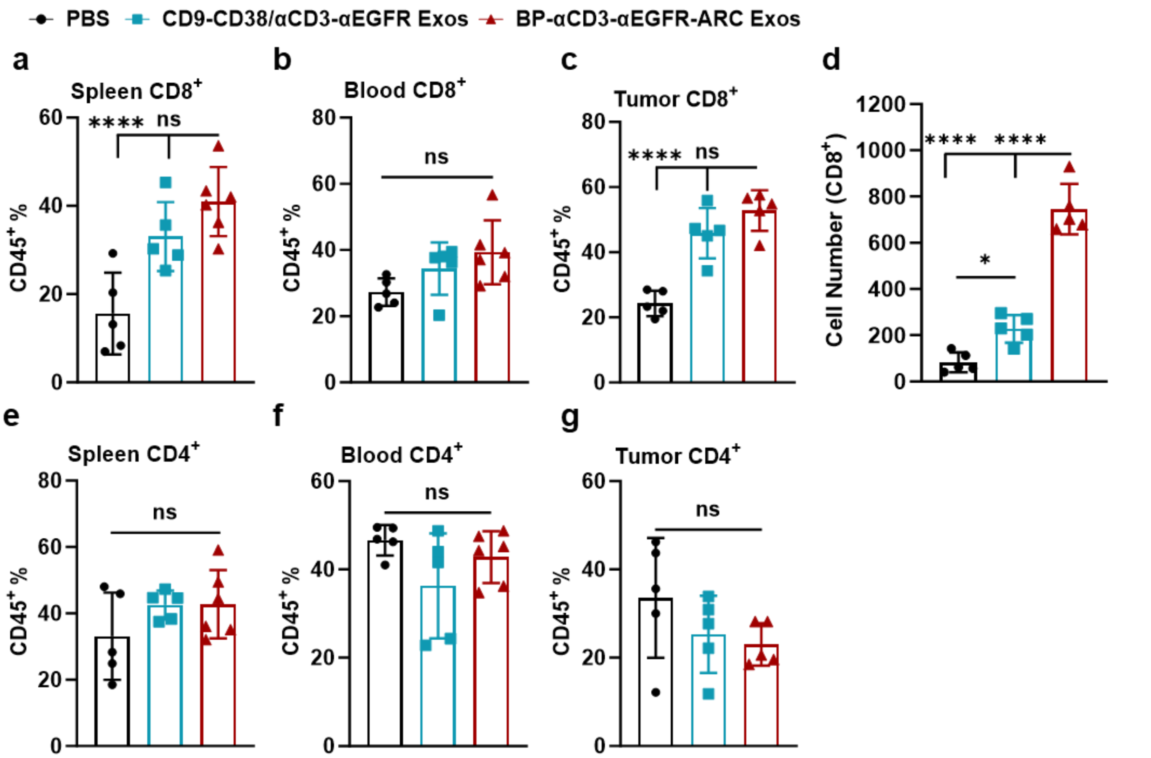

Supplementary Figure 26. T-cell infiltration for MDA-MB-231 tumor-bearing mice at the end of anti-metastasis study by flow cytometry. (a)-(c) Percentages of $CD8^+$ T cells in $CD45^+$ cells in spleens (a), blood (b), and tumors (c) for each group of mice at the end of study (n = 5-6 per group). (d) Quantitative analysis of infiltrating $CD8^+$ T cells in MDA-MB-231 tumors at the end of study (n = 5 per group). (e)-(g) Percentages of $CD4^+$ T cells in $CD45^+$ cells in spleens (e), blood (f), and tumors (g) for each group of mice at the end of study (n = 5 or 6 per group). Data are presented as mean ± SD. Statistical analysis was performed using ordinary one-way ANOVA with Dunnett's multiple comparison test. Significance of finding was defined as follows, ns = not significant $p > 0.05$, * $p < 0.05$, and **** $p < 0.0001$. Source data are provided as a Source Data file.

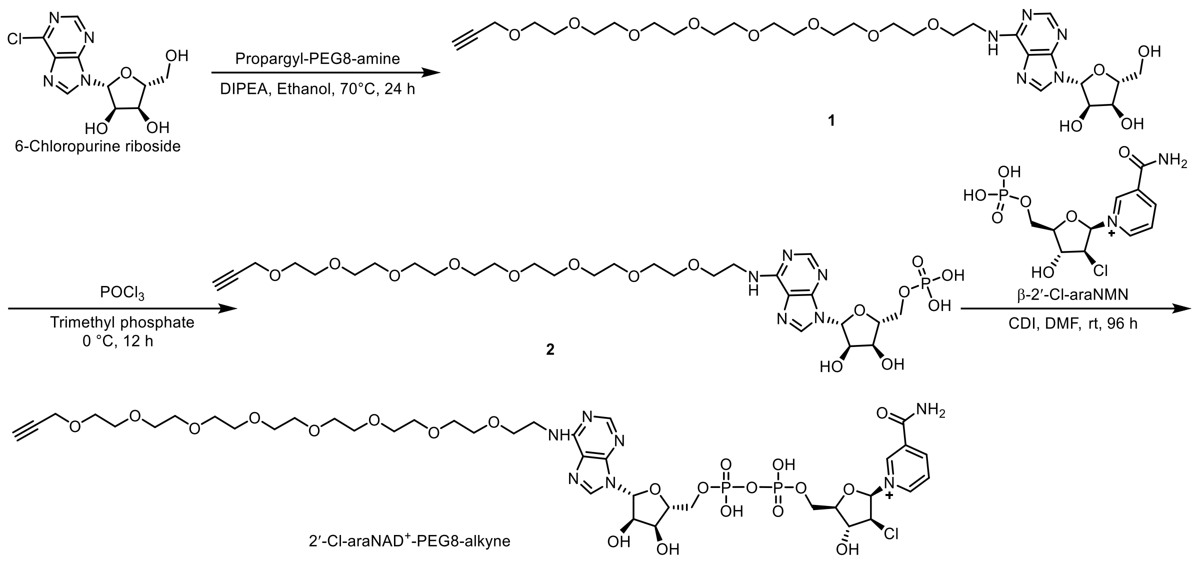

Supplementary Figure 27. Synthetic route for 2′-Cl-araNAD^{+}-PEG8-alkyne.

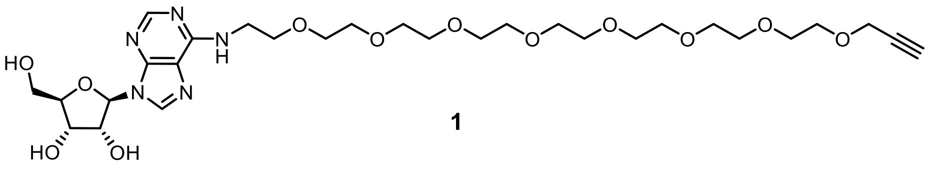

Supplementary Figure 28. Chemical structure of 1.

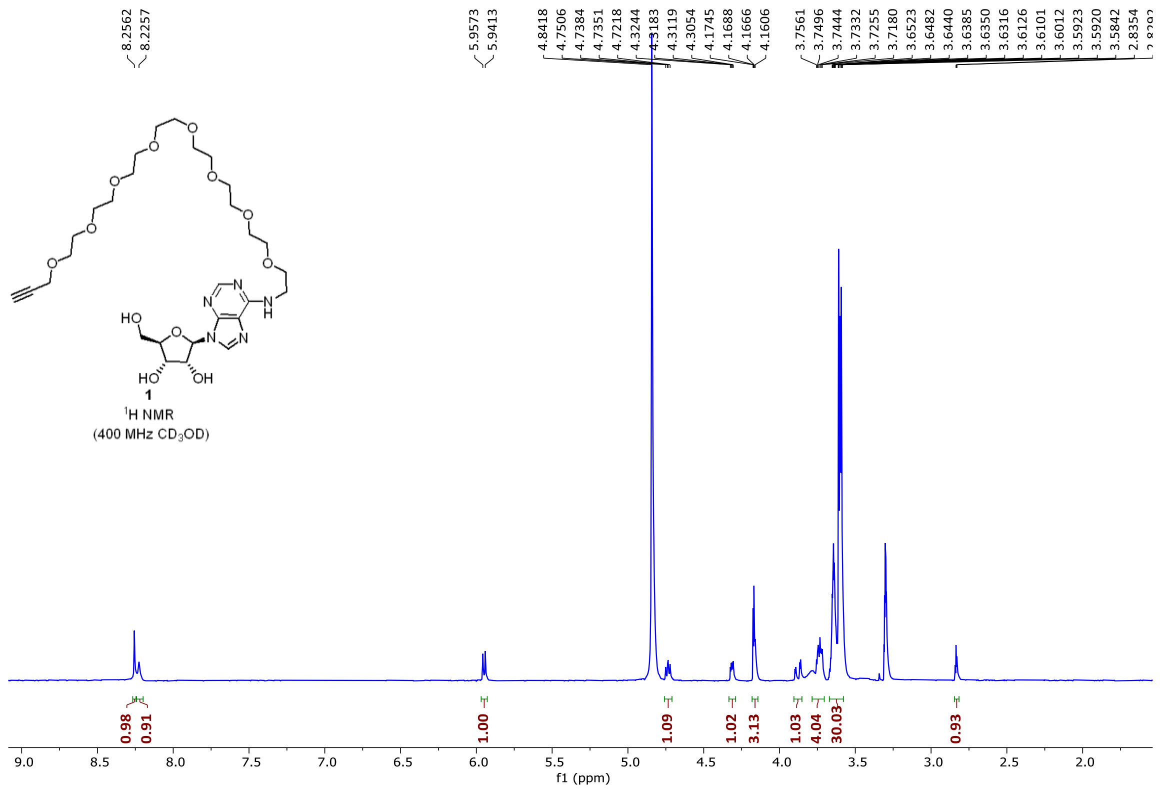

Supplementary Figure 29. ^{1}H NMR spectrum of 1.

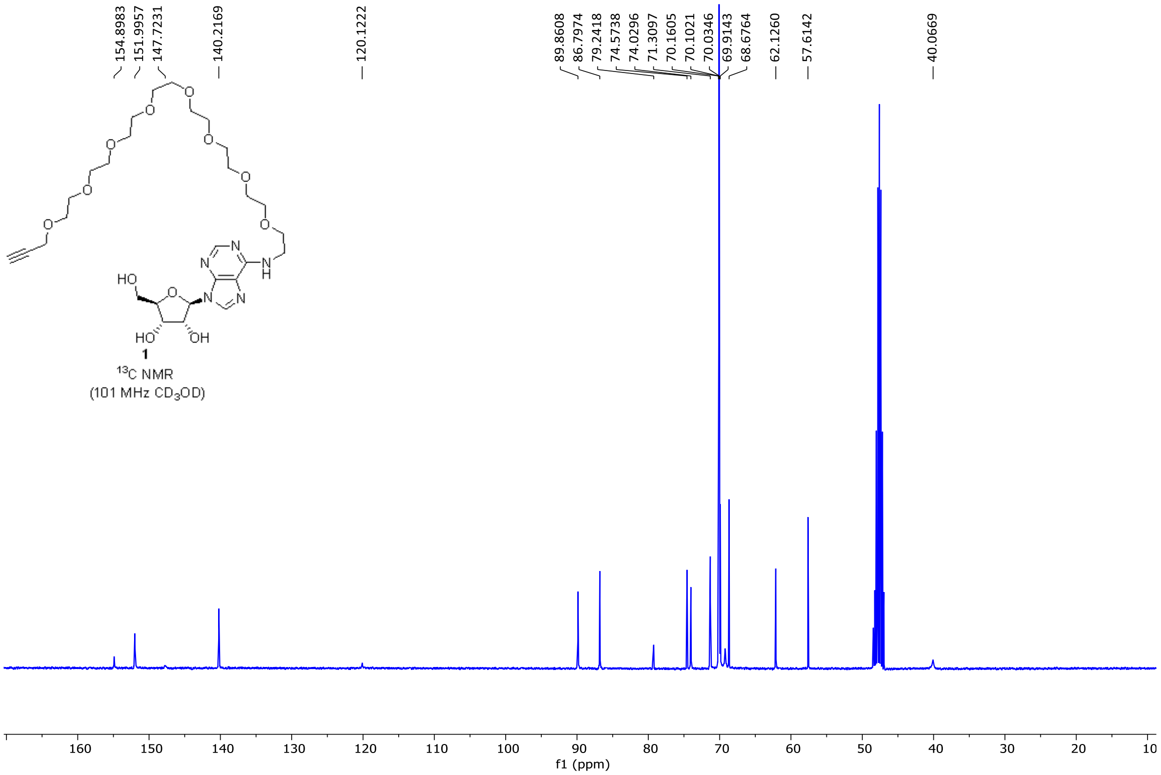

Supplementary Figure 30. ^{13}C NMR spectrum of 1.

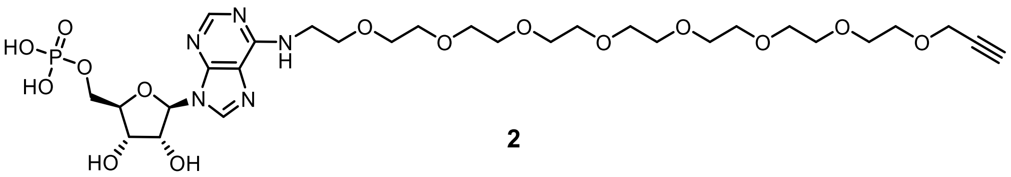

2

Supplementary Figure 31. Chemical structure of 2.

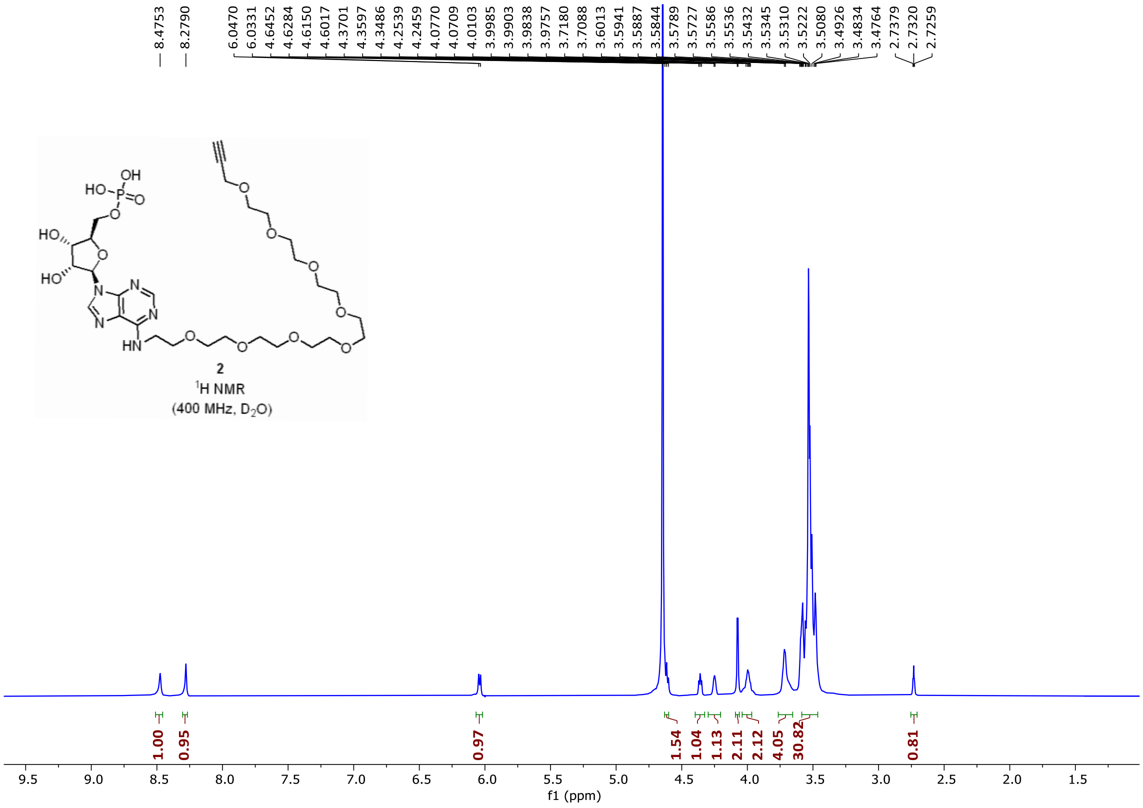

Supplementary Figure 32. ^{1}H NMR spectrum of 2.

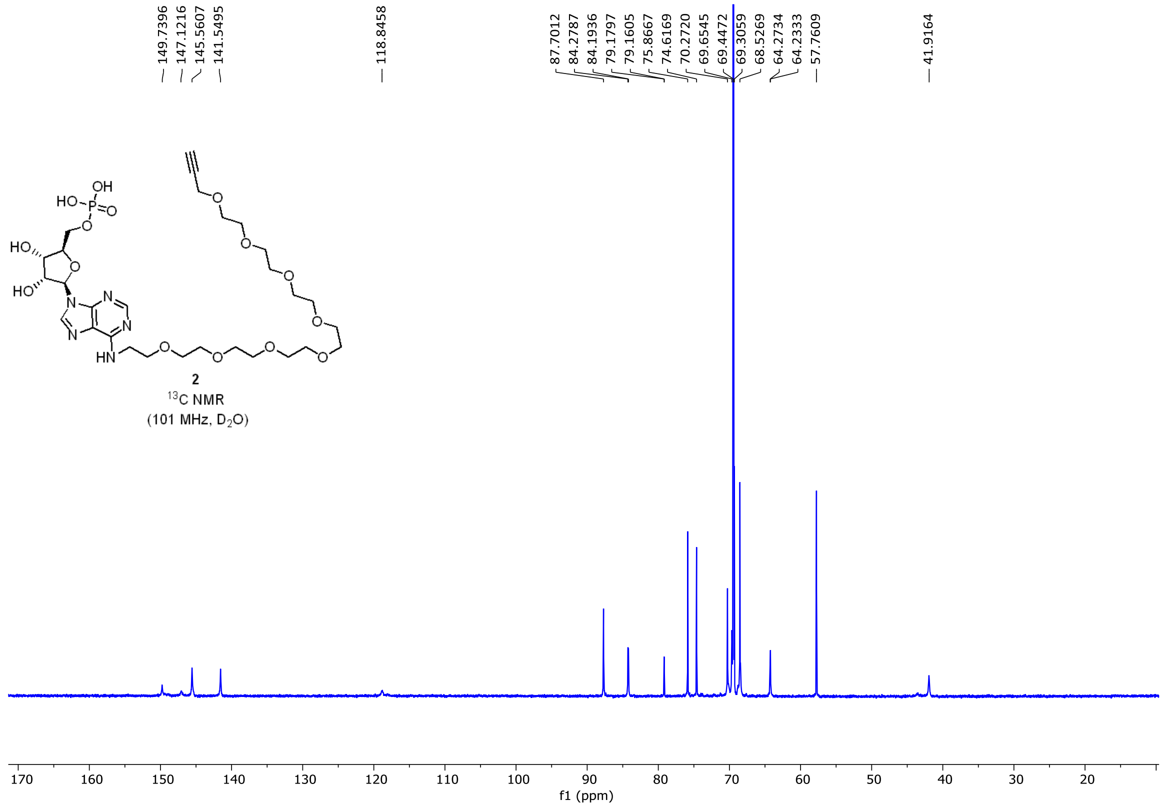

Supplementary Figure 33. ^{13}C NMR spectrum of 2.

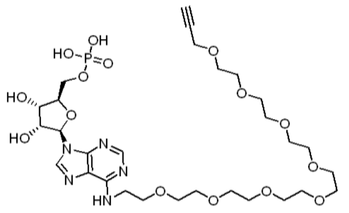

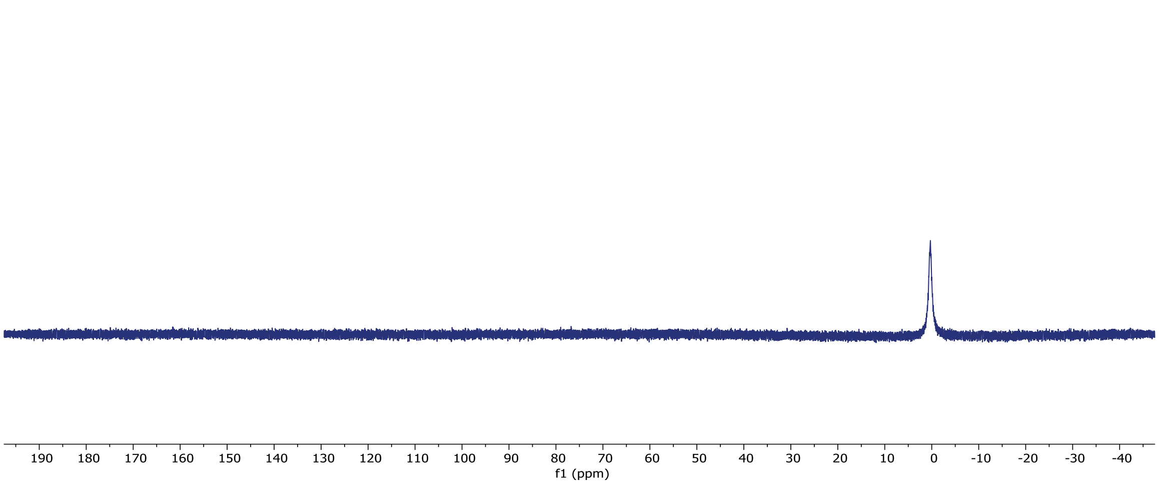

Supplementary Figure 34. ^{31}P NMR spectrum of 2.

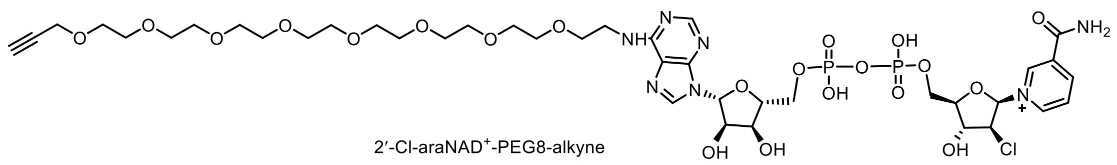

Supplementary Figure 35. Chemical structure of 2′-Cl-araNAD^{+}-PEG8-alkyne.

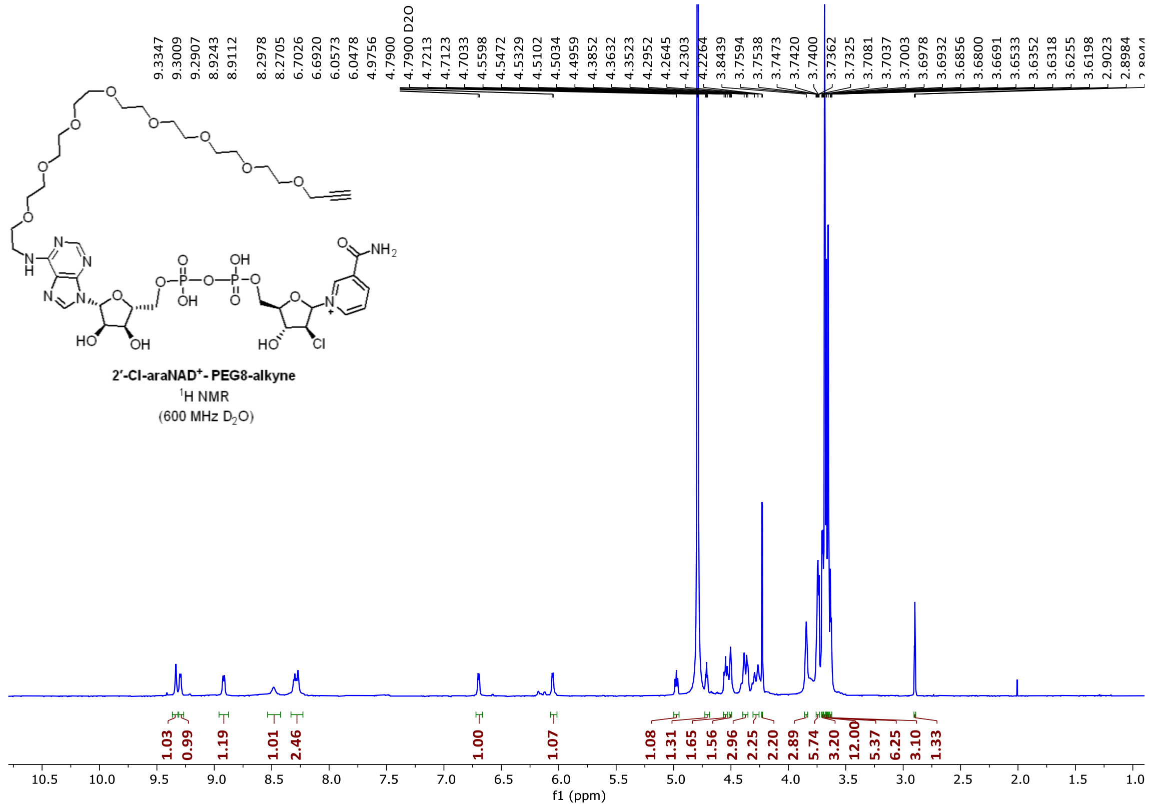

Supplementary Figure 36. ^{1}H NMR spectrum of 2′-Cl-araNAD^{+}-PEG8-alkyne.

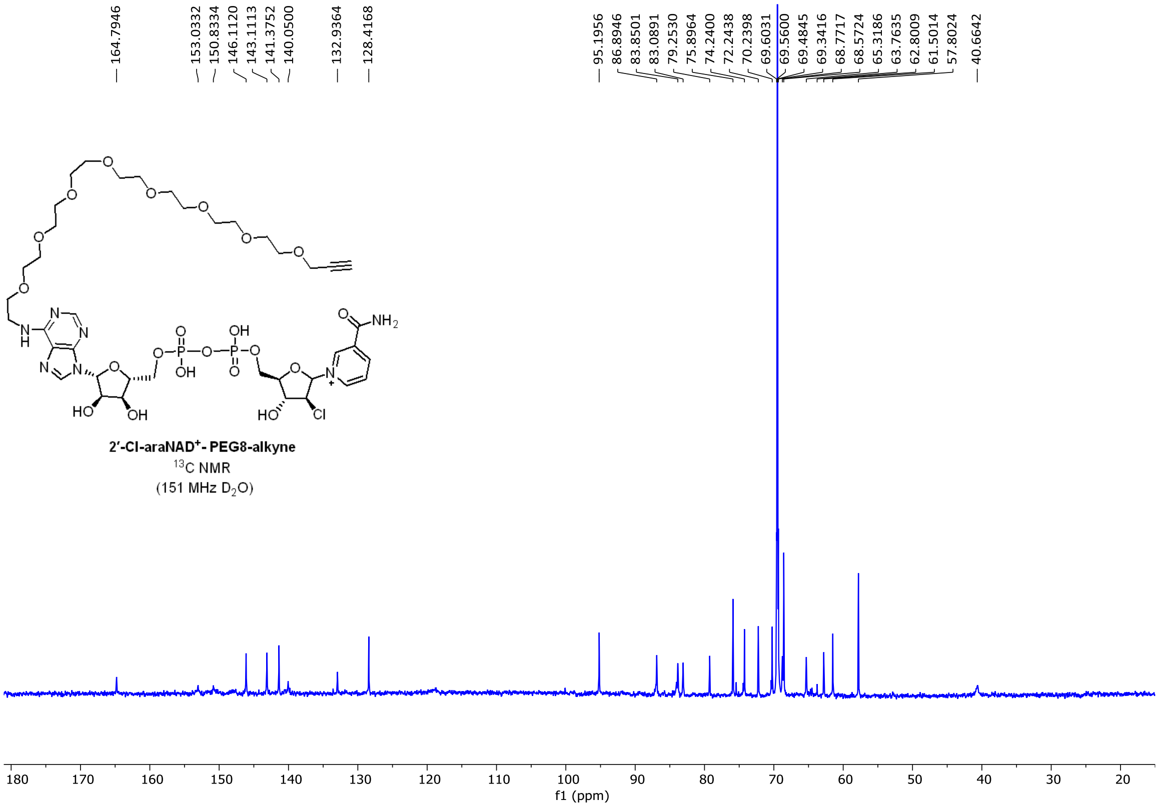

Supplementary Figure 37. ^{13}C NMR spectrum of 2′-Cl-araNAD^{+}-PEG8-alkyne.

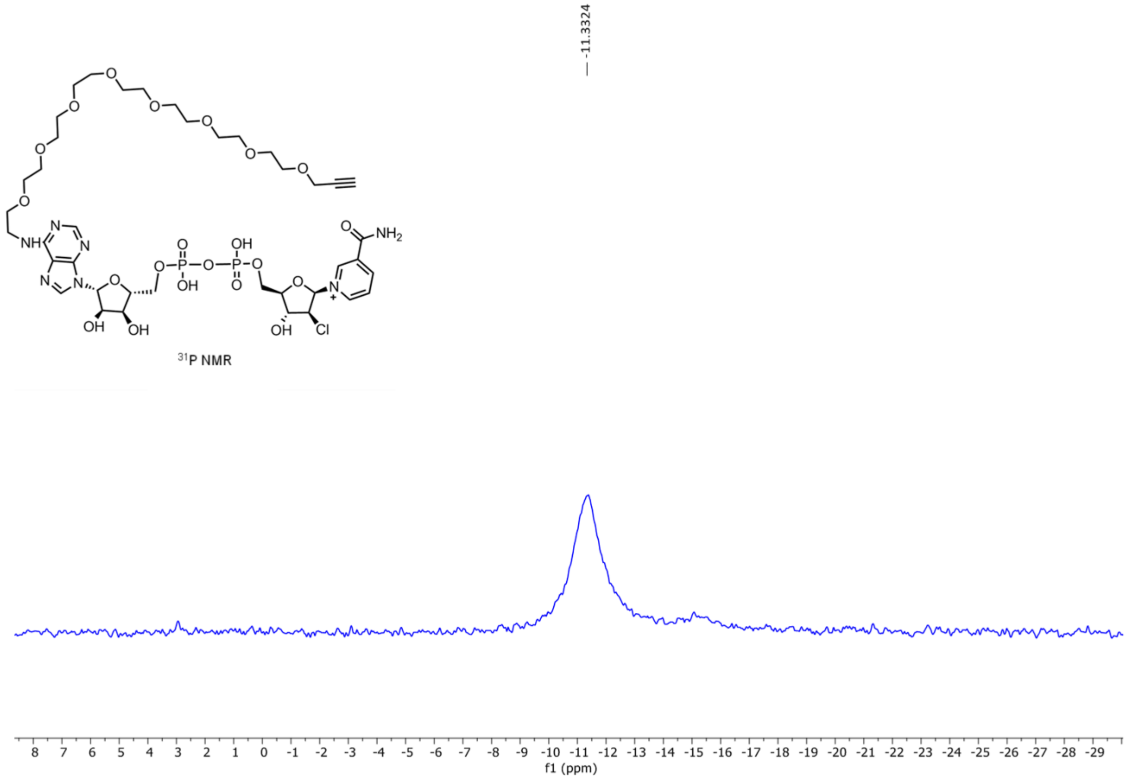

Supplementary Figure 38. ^{31}P NMR spectrum of 2′-Cl-araNAD^{+}-PEG8-alkyne.

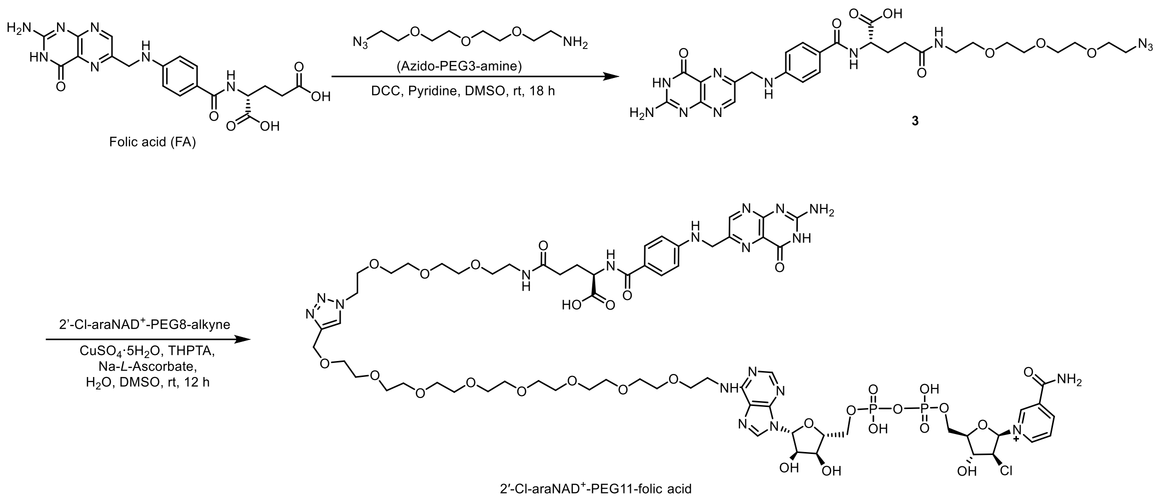

Supplementary Figure 39. Synthetic route for 2′-Cl-araNAD$^+$-PEG11-folic acid.

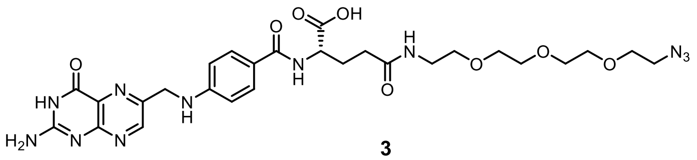

3

Supplementary Figure 40. Chemical structure of 3.

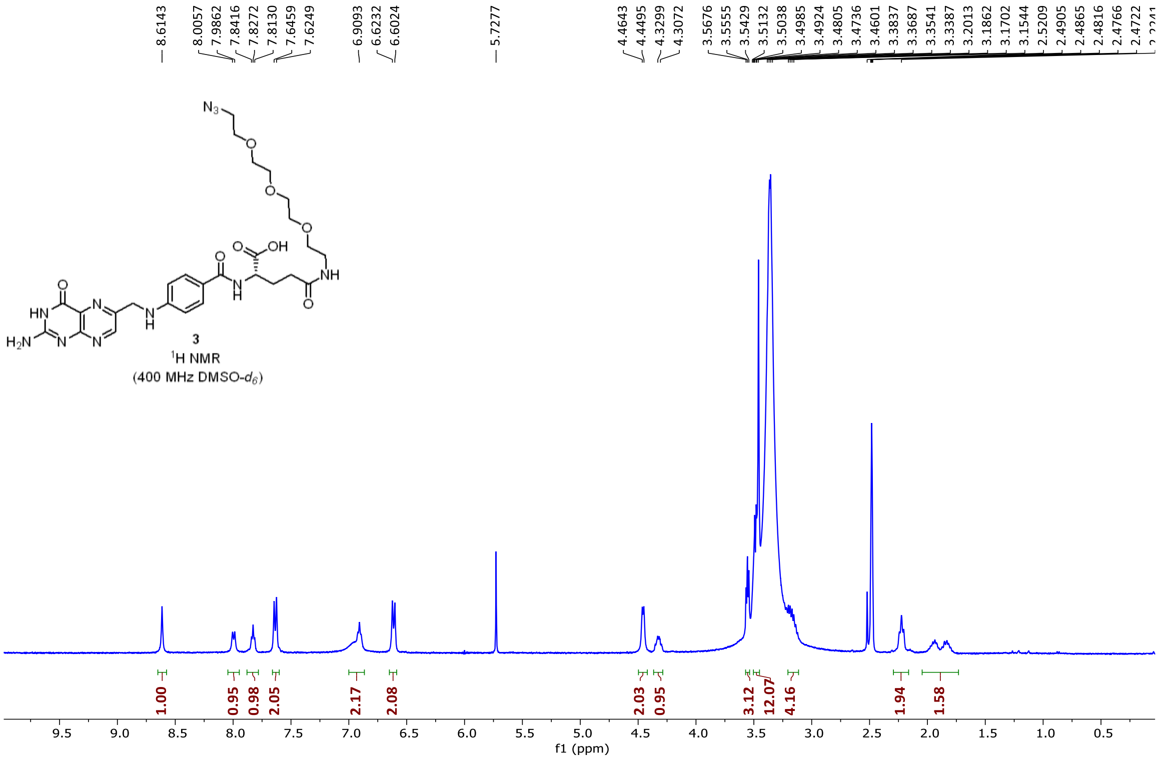

Supplementary Figure 41. ^{1}H NMR spectrum of 3.

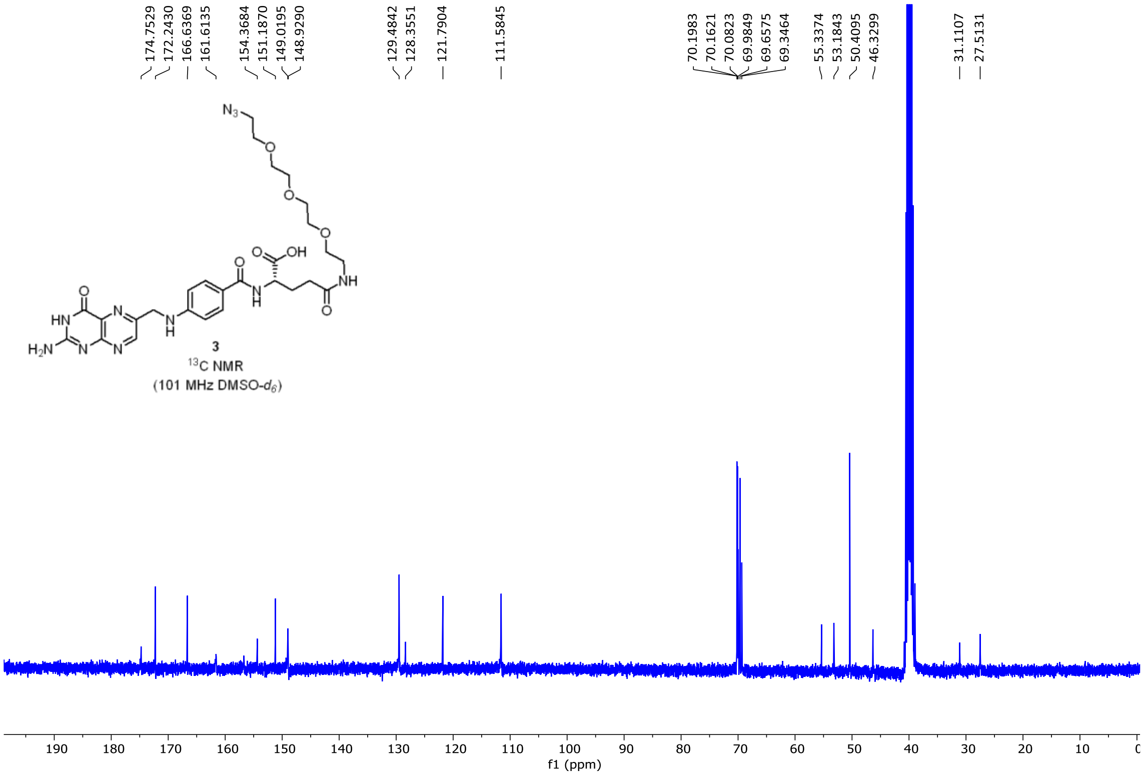

Supplementary Figure 42. ^{13}C NMR spectrum of 3.

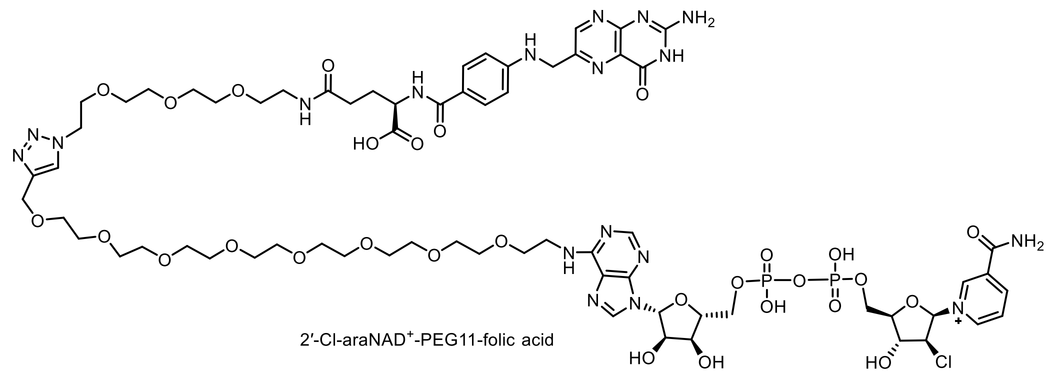

Supplementary Figure 43. Chemical structure of 2′-Cl-araNAD^{+}-PEG11-folic acid.

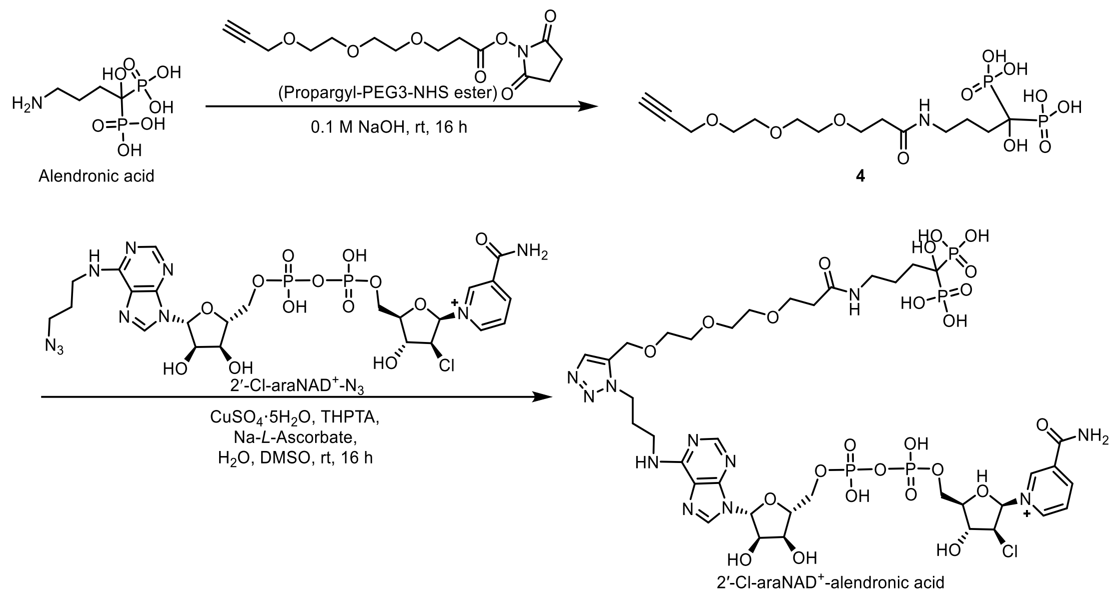

Supplementary Figure 44. Synthetic route for 2′-Cl-araNAD^{+}-alendronic acid.

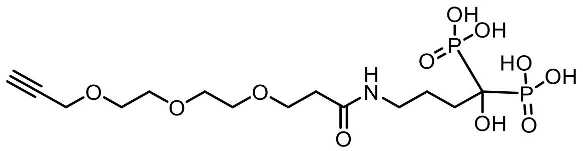

4

Supplementary Figure 45. Chemical structure of 4.

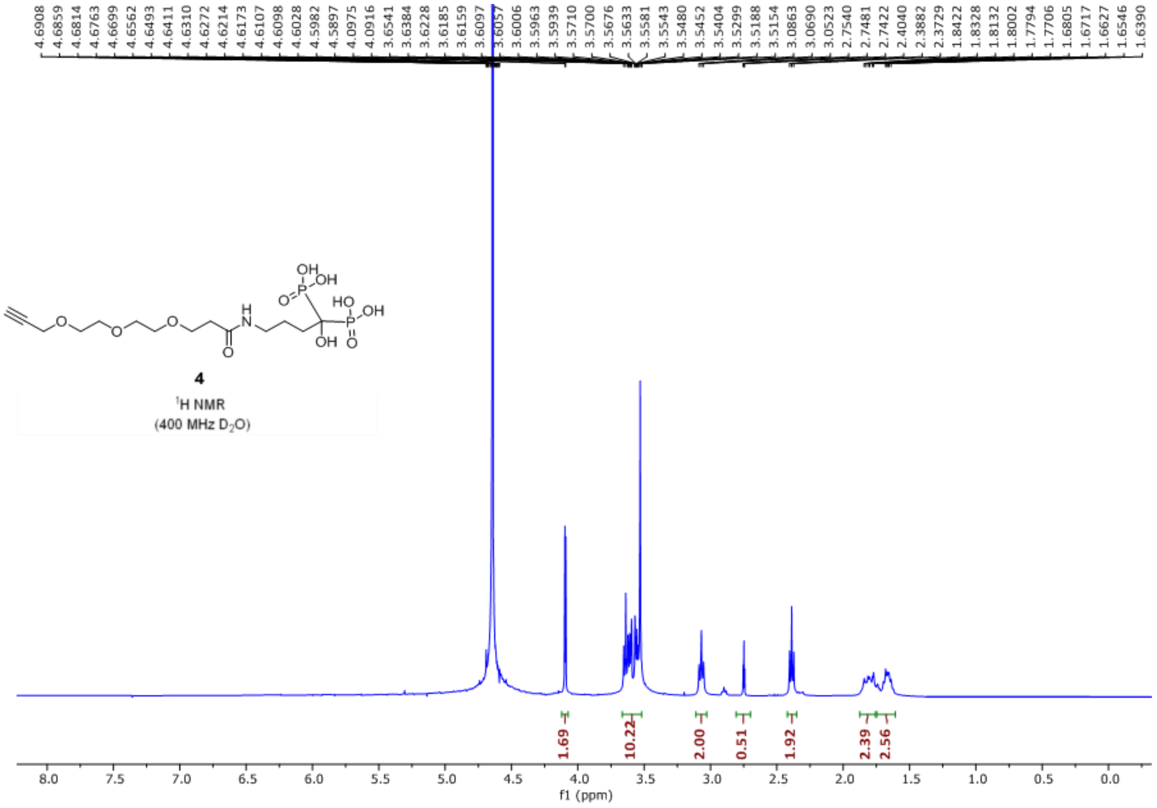

Supplementary Figure 46. ^{1}H NMR spectrum of 4.

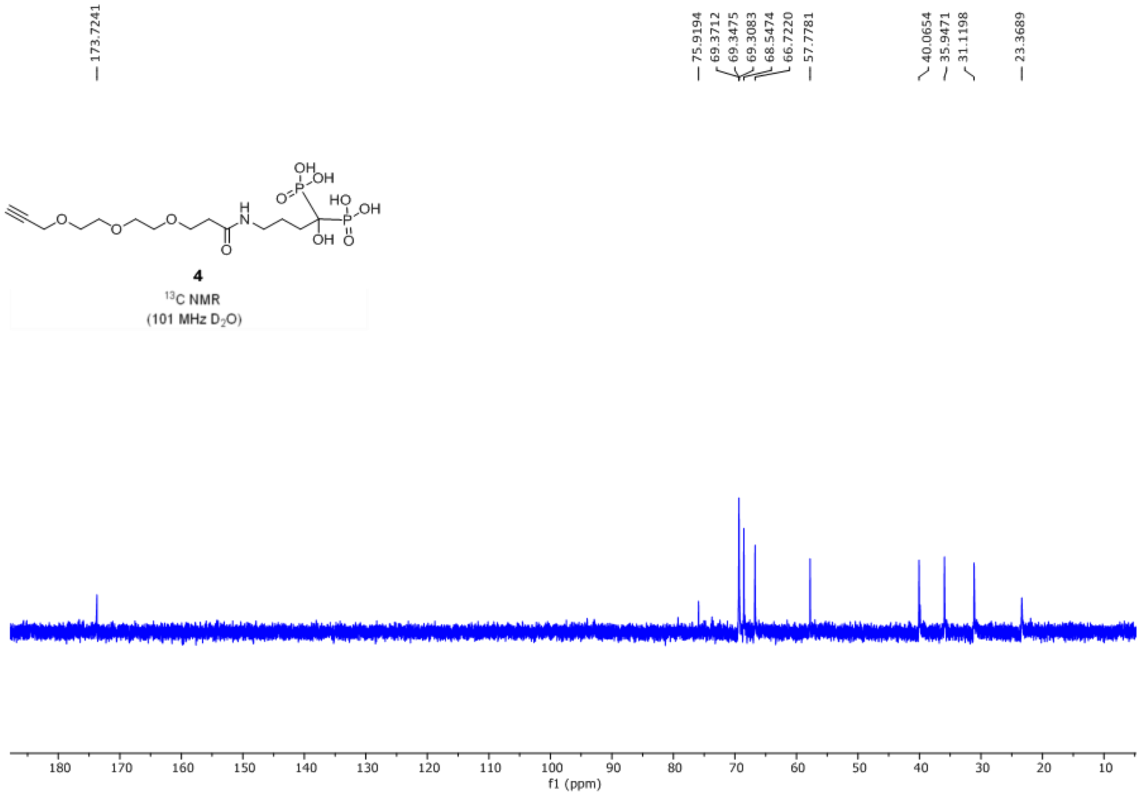

Supplementary Figure 47. ^{13}C NMR spectrum of 4.

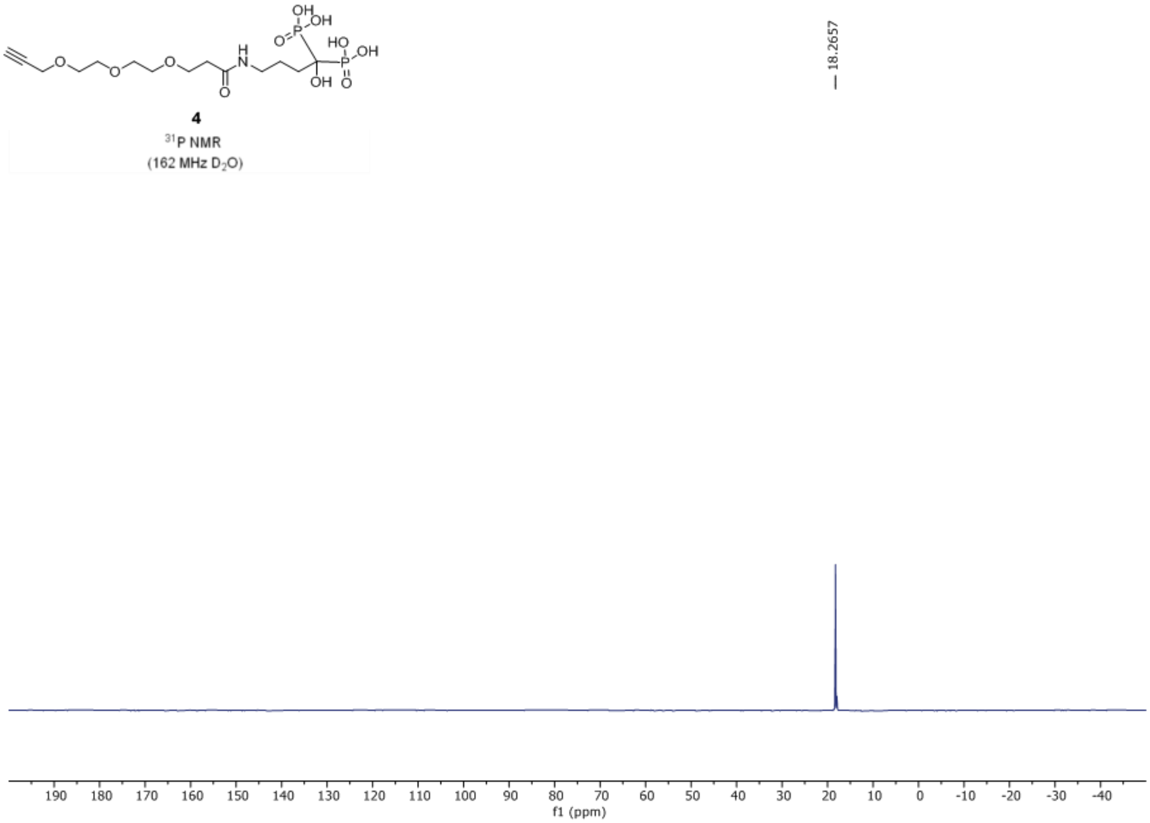

Supplementary Figure 48. ^{31}P NMR spectrum of 4.

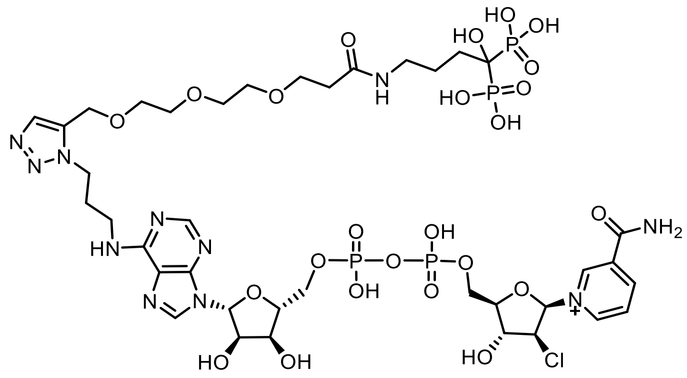

2′-Cl-araNAD^{+}-alendronic acid

Supplementary Figure 49. Chemical structure of 2′-Cl-araNAD^{+}-alendronic acid.

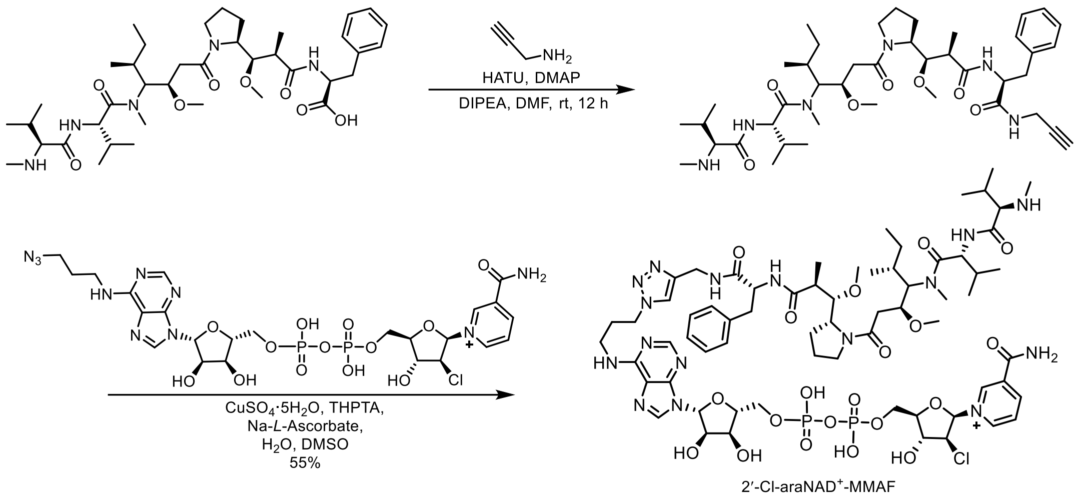

Supplementary Figure 50. Synthetic route for 2′-Cl-araNAD^{+}-MMAF.

Supplementary Method 1. Chemical synthesis of 2′-Cl-araNAD$^+$-derived conjugates

Commercial reagents and solvents were used as received unless otherwise indicated. Reactions were conducted under anhydrous conditions in flasks that were oven-dried for 30 min under dry nitrogen atmosphere. Flash column chromatography was performed using 230-400 mesh silica gel (Sigma-Aldrich, St. Louis, MO). Thin-layer chromatography (TLC) was performed using silica gel plates (Sigma-Aldrich, GF254) and analyzed under 254 nm UV light using diluted samples. High-resolution mass spectra (HRMS) were obtained at the Multi-omics Mass Spectrometry Core of the University of Southern California on a Thermo Fisher Q-Exactive Orbitrap (LC/MS/MS). ^{1}H NMR spectra were recorded on an Oxford AM-400 spectrometer in $CDCl_3$, CD_3OD, or D_2O. The coupling constants, J, are shown in hertz. ^{13}C NMR spectra were recorded using an Oxford AM-400 spectrophotometer (100 MHz) with a complete proton-decoupling spectrophotometer (CD_3OD: 49.0 ppm).

Synthesis of 2′-Cl-araNAD$^+$-PEG8-alkyne

General procedure for the synthesis of **1**. To a solution of 6-chloropurine riboside (200 mg, 0.7 mmol) in ethanol (20.0 mL) were added propargyl-PEG8-amine (200 mg, 0.5 mmol, 0.7 eq) and DIPEA (375 μL, 2 mmol, 3 eq) at room temperature (rt). The resulting mixture was stirred at 70°C for 24 h and the reaction was monitored by TLC. The solvent was next removed under reduced pressure, and the residue was purified by flash column chromatography using 5-10% methanol in dichloromethane as eluent to afford **1** (380 mg, 83%) as a colorless semisolid.

(2R,3R,4S,5R)-2-(6-((3,6,9,12,15,18,21,24-octaoxaheptacos-26-yn-1-yl)amino)-9H-purin-9-yl)-5-(hydroxymethyl)tetrahydrofuran-3,4-diol (1): 380 mg, 83% yield; ^{1}H NMR (400 MHz, CD_3OD): δ 8.26 (s, 1H), 8.23 (s, 1H), 5.95 (d, J = 6.5 Hz, 1H), 4.75-4.72 (m, 1H), 4.31 (dd, J = 5.0, 2.5 Hz, 1H), 4.17 (t, J = 2.8 Hz, 3H), 3.88 (m, 1H), 3.77-3.71 (m, 4H), 3.67-3.58 (m, 34H), 2.84 (t, J = 2.4 Hz, 1H). ^{13}C NMR (101 MHz, CD_3OD): δ 154.90, 152.00, 147.75, 140.22, 120.10, 89.86, 86.80, 79.24, 74.57, 74.02, 71.31, 70.16,

70.10, 70.03, 69.91, 68.68, 62.12, 57.61, 40.09. HRMS (ESI) for $C_{29}H_{48}N_5O_{12}$ $(M+H)^+$: Calcd.: 658.3299; Obs: 658.3281.

General procedure for the synthesis of **2**. To a stirred solution of compound **1** (380 mg, 0.57 mmol) in trimethyl phosphate (5 mL) was added dropwise $P(O)Cl_3$ (107 µL, 1.15 mmol, 2 eq) at 0°C and the resulting mixture was stirred at 0°C for 12 h. H_2O (2 mL) was then added to quench the reaction. The reaction mixture was then extracted with diethyl ether to remove excess trimethyl phosphate. The aqueous layer was purified via HPLC (C18 Kinetex column, 5 µm, 100 Å, 150×10.0 mm, Phenomenex Inc) (mobile phase A: 0.1% formic acid (aq), mobile B: 0.1% formic acid in acetonitrile; flow rate = 2.0 mL min^{-1}; 0-2 min: 0-4% B, 2-4 min: 4-10% B, 4-8 min: 10-20% B, 8-9 min: 20% B, 9-12 min: 20-50% B, 12-14 min: 50-0% B). Fractions containing the desired product were concentrated and lyophilized to yield the desired product compound **2** (250 mg, 58%) as a white semisolid.

((2R,3S,4R,5R)-5-(6-((3,6,9,12,15,18,21,24-octaoxaheptacos-26-yn-1-yl)amino)-9H-purin-9-yl)-3,4-dihydroxytetrahydrofuran-2-yl)methyl dihydrogen phosphate (2): ^1H NMR (400 MHz, D_2O) δ 8.48 (s, 1H), 8.28 (s, 1H), 6.04 (d, J = 5.4 Hz, 1H), 4.39-4.32 (m, 1H), 4.25 (s, 1H), 4.07 (d, J = 2.3 Hz, 2H), 4.00 (s, 2H), 3.72 (s, 4H), 3.60-3.47 (m, 30H), 2.73 (t, J = 2.3 Hz, 1H). ^{13}C NMR (101 MHz, D_2O) δ 149.78, 147.06, 145.56, 141.55, 118.85, 87.70, 84.28, 84.19, 79.18, 79.16, 75.87, 74.62, 70.27, 69.65, 69.45, 69.31, 68.53, 64.27, 64.23 57.76, 41.92. ^{31}P NMR (243 MHz, D_2O) δ 0.32. HRMS (ESI) for $C_{29}H_{49}N_5O_{15}P$ $(M+H)^+$: Calcd.: 738.2963; Obs: 738.2951.

General procedure for the synthesis of 2′-Cl-araNAD$^+$-PEG8-alkyne. To a stirred solution of compound **2** (130 mg, 0.17 mmol) in dried DMF (3 mL) was added 1,1-carbonyldiimidazole (CDI) (142 mg, 0.88 mmol, 5 eq). The reaction mixture was stirred at rt for 14 h and monitored by mass spectroscopy to confirm the formation of intermediate. The reaction mixture then quenched with 0.5 mL dried methanol. The solvent was removed under vacuum, and the residue was co-evaporated three times each with 1 mL of dried DMF. The activated intermediate was dissolved in dried DMF (2 mL) and β-2′-Cl-araNMN

(50 mg, 0.15 mmol, 1.5 eq) was added. After stirring at rt for 4 d, H_2O (20 mL) was added to quench the reaction at 0ºC. The resulting mixture was continued stirring at room temperature for 24 h. The crude product was purified via HPLC using the (C18 Kinetex column, 5 μm, 100 Å, 150×10.0 mm) (mobile phase A: 0.1% formic acid (aq), mobile B: 0.1% formic acid in acetonitrile; flow rate = 2.0 mL min^{-1}; 0-2 min: 0-4% B, 2-4 min: 4-10% B, 4-6 min: 10-20% B, 6-12 min: 20-50% B, 12-17 min: 50-100% B, 17-20 min: 100-0% B) with detection of UV absorbance at 260 nm. Fractions containing the desired product were concentrated and lyophilized to yield 2′-Cl-araNAD$^+$-PEG8-alkyne (29 mg, 41%) as a white solid.

1-((3S,4R,5R)-5-(((((((2R,3S,4R,5R)-5-(6-((3,6,9,12,15,18,21,24-octaoxaheptacos-26-yn-1-yl)amino)-9H-purin-9-yl)-3,4-dihydroxytetrahydrofuran-2-yl)methoxy)(hydroxy)phosphoryl)oxy)(hydroxy)phosphoryl)oxy)methyl)-3-chloro-4-hydroxytetrahydrofuran-2-yl)-3-carbamoylpyridin-1-ium (2′-Cl-araNAD$^+$-PEG8-alkyne): ^{1}H NMR (600 MHz, D_2O) δ 9.33 (s, 1H), 9.30 (d, J = 6.1 Hz, 1H), 8.92 (d, J = 7.8 Hz, 1H), 8.48 (s, 1H), 8.29 (dd, J = 15.3, 8.2 Hz, 2H), 6.70 (d, J = 6.3 Hz, 1H), 6.05 (d, J = 5.7 Hz, 1H), 4.98 (t, J = 7.2 Hz, 1H), 4.71 (t, J = 5.4 Hz, 1H), 4.56-4.53 (m, 2H), 4.51 (d, J = 4.1 Hz, 2H), 4.40-4.35 (m, 3H), 4.28 (d, J = 18.4 Hz, 2H), 4.23 (d, J = 2.3 Hz, 2H), 3.84 (s, 3H), 3.76-3.73 (m, 6H), 3.71-3.70 (m, 3H), 3.68 (d, J = 3.3 Hz, 12H), 3.67 (s, 5H), 3.65 (s, 6H), 3.64-3.62 (m, 3H), 2.90 (t, J = 2.4 Hz, 1H). ^{13}C NMR (151 MHz, D_2O) δ 164.79, 153.03, 150.83, 146.11, 143.11, 141.38, 140.05 132.94, 128.42, 95.20, 86.89, 83.85, 83.09, 79.25, 75.90, 74.24, 72.24, 70.24, 69.60, 69.56, 69.48, 69.34, 68.77, 68.57, 65.32, 63.76, 62.80, 61.50, 57.80, 40.66. ^{31}P NMR (243 MHz, D_2O) δ 11.33. HRMS (ESI) for $C_{40}H_{61}ClN_7O_{21}P_2^+$ $(M)^+$: Calcd.: 1072.3079; Obs: 1072.3042.

Synthesis of 2′-Cl-araNAD$^+$-PEG11-folic acid

General procedure for the synthesis of **3**. In the dark and under a nitrogen atmosphere, to a solution of folic acid (1.00 g, 2.26 mmol) in anhydrous DMSO (40 mL) was added pyridine (20 mL), azido-PEG3-amine (0.543 g, 2.49 mmol), and dicyclohexylcarbodiimide (DCC) (1.17 g, 5.66 mmol) at rt. The reaction mixture was stirred for 12 h at rt. After completion of the reaction (monitored by mass spectroscopy), the reaction mixture was filtered, and

the filtrate was gradually poured into a vigorously stirred solution of diethyl ether (1 L) at 0°C. The yellow precipitate was collected by filtration and the solid compound was washed with acetone and methanol and dried under high vacuum. The crude product was purified via HPLC (C18 Kinetex column, 5 µm, 100 Å, 150×10.0 mm) (mobile phase A: 0.1% formic acid (aq), mobile B: 0.1% formic acid in acetonitrile; flow rate = 2.0 mL min^{-1}; 0-2 min: 0-5% B, 2-4 min: 4-10% B, 4-8 min: 10-30% B, 8-12 min: 30-40% B, 12-20 min: 0-5% B) with detection of UV absorbance at 260 nm. Fractions containing the desired product were concentrated and lyophilized to yield compound **3** (1.3 g, 89%) as a pale yellow solid.

(S)-16-(4-(((2-amino-4-oxo-3,4-dihydropteridin-6-yl)methyl)amino)benzamido)-1-azido-13-oxo-3,6,9-trioxa-12-azaheptadecan-17-oic acid (3): ^{1}H NMR (400 MHz, DMSO-d_6) δ 11.40 (bs, 1H), 8.61 (s, 1H), 7.99 (d, J = 7.4 Hz, 1H), 7.83 (s, 1H), 7.64 (d, J = 8.5 Hz, 2H), 6.96 (s, 1H), 6.91 (m, 2H), 6.61 (d, J = 8.7 Hz, 2H), 4.46 (d, J = 5.3 Hz, 2H), 4.33 (m, 1H), 3.57-3.53 (m, 3H), 3.50-3.48 (m, 4H), 3.46-3.3.44 (3, 5H), 3.19-3.13 (m, 3H), 2.22 (t, 2H), 2.02-1.75 (m, 2H). ^{13}C NMR (DMSO-d_6): 174.74, 172.24, 166.64, 161.61,154.37, 151.19, 148.99, 148.93,129.48, 128.36, 121.79, 111.58, 70.20, 70.16, 70.08, 69.98, 69.66, 69.35, 55.34, 53.18, 50.41, 46.33, 31.13, 27.51. HRMS (ESI) for $C_{27}H_{36}N_{11}O_8$ $(M+H)^+$: Calcd.: 642.2748; Obs: 642.2722.

General procedure for the synthesis of 2′-Cl-araNAD$^+$-PEG11-folic acid. To a solution of 2′-Cl-araNAD$^+$-PEG8-alkyne (3.00 mg, 0.0028 mmol, 1 eq) and $CuSO_4.5H_2O$ (2.79 mg, 0.011 mmol, 4 eq) in H_2O (500 µL) were added a solution of compound **3** (2.69 mg, 0.0042 mmol, 1.5 eq) in DMSO (1 mL), THPTA (2.43 mg, 0.0056 mmol, 2 eq), and sodium-L-ascorbate (4.49 mg, 0.022 mmol, 8 eq) at rt. Then, the reaction mixture was stirred at the same temperature until the reaction was complete (monitored by HPLC). The reaction was purified via HPLC (C18 column, 150×4.6 mm, 5 µm) (mobile phase A: 0.1% formic acid (aq), mobile B: 0.1% formic acid in acetonitrile; flow rate = 1.0 mL min^{-1}; 0-2 min: 0-4% B, 2-4 min: 4-10% B, 4-6 min: 10-20% B, 6-12 min: 20-50% B, 12-17 min: 50-100% B, 17-20 min: 100-0% B) with detection of UV absorbance at 260 nm. Fractions containing

the desired product were concentrated and lyophilized to yield the 2′-Cl-araNAD$^+$-PEG11-folic acid (2.3 mg, 48%) as a colorless solid.

1-((3S,4R,5R)-5-((((((((2R,3S,4R,5R)-5-(6-((1-(1-((S)-1-(4-(((2-amino-4-oxo-3,4-dihydropteridin-6-yl)methyl)amino)phenyl)-3-carboxy-1,6-dioxo-10,13,16-trioxa-2,7-diazaoctadecan-18-yl)-1H-1,2,3-triazol-4-yl)-2,5,8,11,14,17,20,23-octaoxapentacosan-25-yl)amino)-9H-purin-9-yl)-3,4-dihydroxytetrahydrofuran-2-yl)methoxy)(hydroxy)phosphoryl)oxy)(hydroxy)phosphoryl)oxy)methyl)-3-chloro-4-hydroxytetrahydrofuran-2-yl)-3-carbamoylpyridin-1-ium (2′-Cl-araNAD$^+$-PEG11-folic acid): HRMS (ESI) for $C_{67}H_{96}ClN_{18}O_{29}P_2^+$ $(M+H)^+$: Calcd: 857.2913; Obs: 857.2896.

3. Synthesis of 2′-Cl-araNAD$^+$-alendronic acid.

General procedure for the synthesis of **4**. To a stirred solution of alendronic acid (100 mg, 0.40 mmol) in Milli-Q water (2 mL) was added 0.1 M NaOH till reaching pH 8.5, forming a clear solution. To the reaction mixture was added propargyl-PEG3-NHS ester (1.2 eq) in acetonitrile (2 mL) portion wise by re-adjusting pH to 8.5 with 0.1 N NaOH (forming a clear solution). The reaction mixture was stirred at rt overnight, concentrated to a minimum amount, and 10% methanol in the diethyl ether was added. The precipitated solid compound was filtered and dissolved in a minimum amount of Milli-Q water and purified by HPLC (C18-300A column, 250×4.60 mm, 5 μm) (mobile phase A: 0.1% formic acid (aq), mobile B: 0.1% formic acid in methanol; flow rate = 1.0 mL min^{-1}; 0-4 min: 10-22% B, 4-8 min: 22-32% B, 8-12 min: 10-22% B) with detection of UV absorbance at 260 nm. The fractions containing the desired product were concentrated and lyophilized to yield compound **4** (156 mg, 87%) as a white solid.

(18-hydroxy-13-oxo-4,7,10-trioxa-14-azaoctadec-1-yne-18,18-diyl)bis(phosphonic acid) (4): ^{1}H NMR (400 MHz, D_2O) δ 4.09 (d, J = 2.4 Hz, 2H), 3.68-3.50 (m, 10H), 3.07 (t, J = 6.8 Hz, 2H), 2.75 (t, J = 2.4 Hz, 1H, Alkyne), 2.39 (t, J = 6.2 Hz, 2H), 1.89-1.73 (m, 2H), 1.67 (dt, J = 9.2, 6.5 Hz, 2H). ^{13}C NMR (101 MHz, D_2O) δ 173.85, 75.91, 70.04, 69.37, 69.34, 69.30, 68.54, 66.72, 57.77, 40.06, 35.94, 31.11, 23.36. ^{31}P NMR (162 MHz,

D_2O) δ 18.26, 17.94. HRMS (ESI) for $C_{14}H_{28}NO_{11}P_2$ $(M+Na)^+$: Calcd.: 470.0957; Obs: 470.0950.

General procedure for the synthesis of 2′-Cl-araNAD$^+$-alendronic acid. To a solution of compound **4** (3.00 mg, 0.0067 mmol) and $CuSO_4.5H_2O$ (6.68 mg, 0.026 mmol, 4 eq) in H_2O (500 µL) were added a solution of 2′-Cl-araNAD$^+$-N_3 (6.68 mg, 0.0067 mmol, 1.0 eq), THPTA (0.29 mg, 0.00067 mmol, 0.1 eq), and sodium-L-ascorbate (10.6 mg, 0.054 mmol, 8 eq) at rt. Then, the reaction mixture was stirred at the same temperature until the reaction completed. The reaction was purified via HPLC (C18-A column, 150×4.6 mm, 5 µm) (mobile phase A: 0.1% formic acid (aq), mobile B: 0.1% formic acid in acetonitrile; flow rate = 1.0 mL min^{-1}; 0-2 min: 0- 4% B, 2-4 min: 4-10% B, 4-6 min: 10-20% B, 6-12 min: 20-50% B, 12-17 min: 50-100% B, 17-20 min: 100-0% B) with detection of UV absorbance at 260 nm. Fractions containing the desired product were concentrated and lyophilized to yield the 2′-Cl-araNAD$^+$-alendronic acid (3.1 mg, 38%) as a colorless solid. HRMS (ESI) for $C_{38}H_{59}ClN_{11}O_{24}P_4^+$ $(M+H)^+/2$ Calcd.: 606.6223; Obs: 606.6198.

Synthesis of 2′-Cl-araNAD$^+$-MMAF

General procedure for the synthesis of alkynyl-MMAF. Propargyl amine (3.3 mg, 10 eq) and DIPEA (3 µL, 3 eq) in 0.5 mL of DMF was added into 0.5 mL of DMF with MMAF (4.4 mg), HATU (3.0 mg, 1.3 eq), and DMAP (0.2 mg, 0.3 eq) and stirred beginning at 0°C to room temperature. Upon completion of the reaction, alkynyl-MMAF was purified by HPLC as previously reported[1]. The yield of alkynyl-MMAF was 54% and its purity was 98% based on HPLC analysis.

General procedure for the synthesis of 2′-Cl-araNAD$^+$-MMAF. Alkynyl-MMAF (9.2 mg, 1.2 eq), THPTA (86.9 mg, 20 eq), and sodium-*L*-ascorbate (63.4 mg, 32 eq) in 0.2 mL of DMSO was added into 0.5 mL of H_2O with 2′-Cl-araNAD$^+$-N_3 (7.7 mg) and $CuSO_4 \cdot 5H_2O$ (10.0 mg, 4 eq) and stirred at room temperature. Upon completion of the reaction, 2′-Cl-araNAD$^+$-MMAF was purified by HPLC as previously described[1]. The yield of 2′-Cl-araNAD$^+$-MMAF was 55% and its purity was 98% according to HPLC analysis.

Supplementary reference

1. Dai, Z. *et al.* Synthesis of site-specific antibody-drug conjugates by ADP-ribosyl cyclases. *Sci. Adv.* **6**, eaba6752(2020).
